# An adenosine derivative promotes mitochondrial supercomplexes reorganization and restoration of mitochondria structure and bioenergetics in a diethylnitrosamine-induced hepatocellular carcinoma model

**DOI:** 10.1038/s41598-024-56306-9

**Published:** 2024-03-15

**Authors:** Rosendo García-Carrillo, Francisco A. Molina-Pelayo, David Zarate-Lopez, Alejandro Cabrera-Aguilar, Bibiana Ortega-Domínguez, Mariana Domínguez-López, Natalia Chiquete-Félix, Adan Dagnino-Acosta, Gabriela Velasco-Loyden, Enrique Chávez, Luis Castro-Sánchez, Victoria Chagoya de Sánchez

**Affiliations:** 1 Centro Universitario de Investigaciones Biomédicas, Universidad de Colima, 28045 Colima, México; 2 Departamento de Biología Celular y Desarrollo, Instituto de Fisiología Celular, Universidad Nacional Autónoma de México, 04510 Ciudad de México, México; 3 Departamento de Genética Molecular, Instituto de Fisiología Celular, Universidad Nacional Autónoma de México, 04510 Ciudad de México, México; 4 CONAHCYT-Universidad de Colima, 28045 Colima, México

**Keywords:** Cancer, Cell biology

## Abstract

Hepatocellular carcinoma (HCC) progression is associated with dysfunctional mitochondria and bioenergetics impairment. However, no data about the relationship between mitochondrial supercomplexes (hmwSC) formation and ATP production rates in HCC are available. Our group has developed an adenosine derivative, IFC-305, which improves mitochondrial function, and it has been proposed as a therapeutic candidate for HCC. We aimed to determine the role of IFC-305 on both mitochondrial structure and bioenergetics in a sequential cirrhosis-HCC model in rats. Our results showed that IFC-305 administration decreased the number and size of liver tumors, reduced the expression of tumoral markers, and reestablished the typical architecture of the hepatic parenchyma. The livers of treated rats showed a reduction of mitochondria number, recovery of the mtDNA/nDNA ratio, and mitochondrial length. Also, IFC-305 increased cardiolipin and phosphatidylcholine levels and promoted hmwSC reorganization with changes in the expression levels of hmwSC assembly-related genes. IFC-305 in HCC modified the expression of several genes encoding elements of electron transport chain complexes and increased the ATP levels by recovering the complex I, III, and V activity. We propose that IFC-305 restores the mitochondrial bioenergetics in HCC by normalizing the quantity, morphology, and function of mitochondria, possibly as part of its hepatic restorative effect.

## Introduction

Hepatocellular carcinoma (HCC) is the most common hepatic neoplasm (83% of the cases reported). Due to its high prevalence and substantial mortality rate, this cancer represents a significant health challenge^1^; hence, novel treatment and diagnostic strategies are an ongoing priority. Our group developed an adenosine derivative, adenosine conjugated with aspartic acid (IFC-305), that has shown therapeutic effects by reversing the hepatic fibrogenesis and the chemical hepatocarcinogenesis induced by carbon tetrachloride (CCl_4_)^2^ and diethylnitrosamine (DEN)^3^, respectively. This compound reduces the number and tumor size by decreasing the cell proliferation and down-regulating the expression of some factors such as the proliferating cell nuclear antigen (PCNA), thymidylate synthase, HGF, and its receptor c-Met; conversely, it up-regulates the expression of the cell cycle inhibitor protein p27^3,4^, suggesting that IFC-305 prevents hepatic tumorigenesis^3^. Besides, IFC-305 improves mitochondrial dynamics in a sequential model of cirrhosis-HCC induced by DEN^5^.

Mitochondria is an abundant organelle in liver that plays an essential role in the HCC progression^6^ by regulating processes such as the redox state^7^, metabolism^8^, and cell death pathways^9^. Several mitochondrial alterations have been described in HCC, including an increased fission rate, decrease of ATP production by mitochondria in hepatocytes^6^ and decreased mitochondrial DNA (mtDNA) content^10^; moreover, the increase in mitochondrial biogenesis occurs with accumulation of non-functional mitochondria^11^ and deterioration of mitochondrial bioenergetics by diminished activity of the electron transport chain (ETC) complexes I^12^ and III^13^. In HCC, metabolic reprogramming allows energy production through the glycolytic pathway and oxidative phosphorylation (OXPHOS). Until now, identifying priority energy pathways in HCC development has been controversial^14,15^; hence, regulating energy production is a topic to attend.

Adequate mitochondrial function is associated with a quality control process called mitochondrial dynamics that includes fission and fusion; however, when mitochondria are exposed to stress or harmful agents, fission predominates over fusion^5^. Fission involves the separation of mitochondrion into two fractions, a non-functional or damaged fraction and another one with conventional mitochondrial membrane potential (ψm), adequate formation of mitochondrial cristae, and normal ATP production^16^. Mitochondrial morphology is required for regular bioenergetic performance of this organelle; therefore, structural components such as cardiolipin, prohibitin, dimers of complex V of the ETC, high molecular weight mitochondrial supercomplexes (hmwSC) of type I/III_2_/IV_n_, also known as respirosome, maintain the mitochondrial architecture. Previous reports have suggested that assembly of SC is plastic and, in several disease models, is associated with increased efficiency of mitochondrial energy production, facilitates electron transport, and reduces reactive oxygen species (ROS)^17–21^. Factors such as COX7A2L, UQCC3, and HIGD2A, and the preservation of OPA1 levels by the proteases iAAA (YME1L) and mAAA (Paraplegin)^20^ contribute to the assembly of hmwSC and the maintenance of the mitochondrial cristae structure^5,21^.

The mitochondrial fission rate and decreasing the autophagic flux of mitochondria (mitophagy) in HCC prevent the mitochondrial repair process, a part of the mitochondrial quality control mechanism; this promotes the accumulation of non-functional mitochondria. IFC-305 restores mitophagy, suggesting that this process plays a role in the liver restoration effect in the sequential cirrhosis-HCC model^22^. It is known that energy production is reduced in HCC; nevertheless, there is a lack of in vitro or in vivo data on the hmwSC assembly and association with structural components of mitochondria in this context. Since IFC-305 has liver restorative and protective properties and is a promising antitumoral compound for HCC treatment, this work aims to determine the changes in the assembly of the hmwSC and their relationship with modifications of mitochondrial bioenergetic in HCC and finally evaluate the compound IFC-305 restoring these alterations in a sequential cirrhosis-HCC model.

## Results

### IFC-305 downregulates the HCC markers in the experimental model induced by DEN

**Figure 1:**
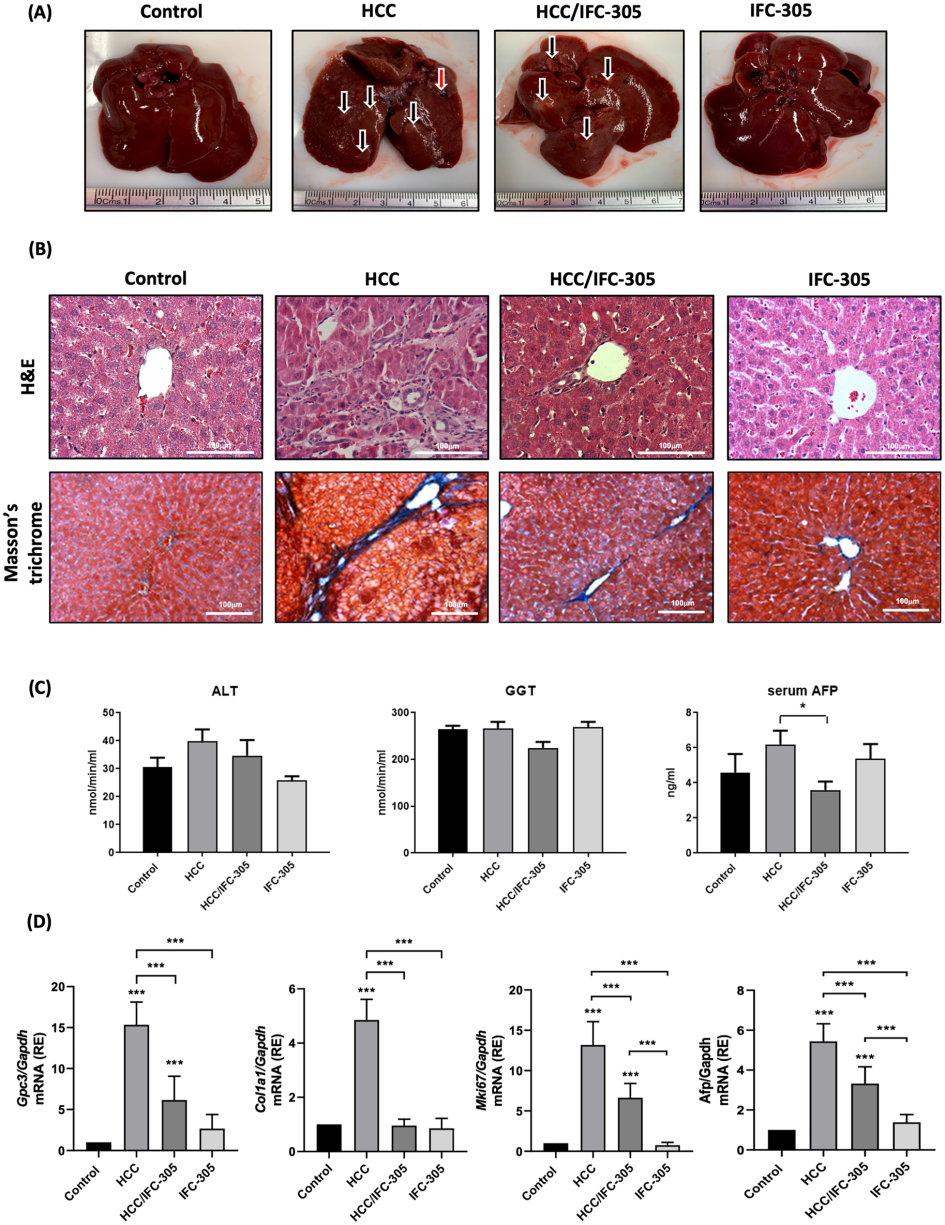
Effects of IFC-305 in sequential cirrhosis-hepatocellular carcinoma model in rats. (**A**) Representative images of liver tissue correspond to the different experimental groups; black arrows represent hepatic nodules, and the red arrow indicates a tumor. (**B**) H&E (upper panel) and Masson's trichrome (lower panel) staining representative images. (**C**) Biochemical markers for liver damage include ALT, GGT, and serum AFP levels (n = 5 per group). (**D**) Determination of mRNA expression levels by RT-qPCR by the comparative Ct method for *Gpc3*, *Col1a1, Mki67*, and *Afp* genes (n = 5 per group). Data are means ± SEM of the five animals per group analyzed by triplicate. One-way ANOVA with Bonferroni test as post hoc *P < 0.05, ** *P* < 0.01, and *** *P* < 0.001.

A sequential model of cirrhosis-HCC was developed to validate the antitumorigenic activity of the IFC-305 and evaluate its effect on mitochondrial structure and bioenergetics. The animals were divided into four experimental groups: Control, HCC, HCC/IFC-305, and IFC-305 (Supplementary Fig. 1). Figure 1A shows a representative liver for each experimental group; liver slices stained with hematoxylin–eosin showed cytological atypia, morphologic polymorphism with a solid growth pattern, and giant cell formations in the HCC condition (Fig. 1B,) in contrast with typical hepatic tissue architecture of the control condition, HCC/IFC-305, and IFC-305 groups; also, IFC-305 treatment reduced the collagen deposition observed in HCC group (Fig. 1B, lower panel). In addition, hepatic damage was determined by the serum enzyme activity of GGT and ALT, and for determining the HCC development, AFP levels were measured (Fig. 1C); ALT and GGT activities showed a tendency to increase with no significant differences in the HCC group when compared with the control group; likewise, the HCC/IFC-305 and IFC-305 groups do not show changes compared to the control group. In contrast, serum AFP levels were elevated in the HCC group but decreased in the HCC/IFC-305 condition. Furthermore, the expression levels of the HCC clinical molecular markers *Gpc3* and *Afp*, the proliferation marker *Mki67*, and the fibrosis marker *Col1a1* were determined by RT-qPCR (Fig. 1D); the results showed a significantly increased expression of all genes determined in the HCC group when compared with the control group; moreover, the administration of IFC-305 reversed this effect. Finally, the IFC-305 group showed similar levels compared to the control condition.

### IFC-305 promotes recovery of the mitochondrial structure in the cirrhosis-HCC sequential model

**Figure 2:**
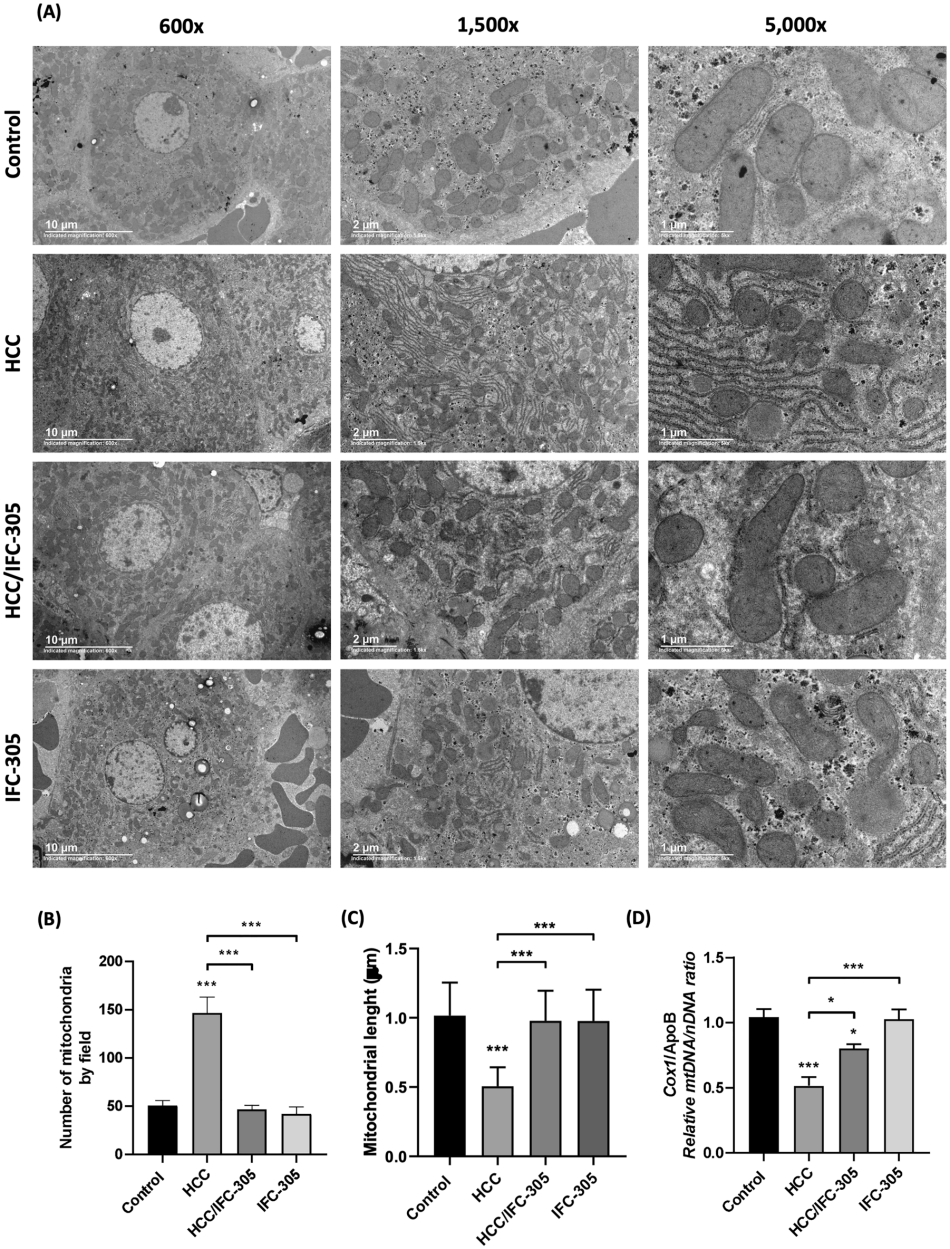
Mitochondrial structure by transmission electron microscopy. (**A**) The panel shows micrographs of liver tissue from the different experimental groups obtained by transmission electron microscopy; the exposed fields show the mitochondria present in the hepatocytes using magnifications 600 x, 1,500 x, and 5,000 x. (**B**) The number of mitochondria evaluated in 3 fields per rat in each condition (n = 5 per group). (**C**) Graphical representation of the mitochondrial length in micrometers of the different treatment schemes (n = 5 per group) and (**D**) Determination of mtDNA/nDNA ratio by RT-qPCR by the comparative Ct method for *Cox1* and *ApoB* (n = 5 per group). Data are means ± SEM of the five animals per group analyzed by triplicate; One-way ANOVA with Bonferroni test as post hoc * *P* < 0.05, ** *P* < 0.01, and *** *P* < 0.001.

Once the HCC experimental model was molecular and biochemically validated with HCC markers, we evaluated the mitochondrial structure from the liver in the different experimental groups (mitochondrial fraction purity is shown in supplementary Fig. 2). Transmission electron microscopy (TEM) results showed that control group maintained an enlarged and homogeneous distribution of mitochondria in the hepatocyte and mitochondrial lengths ranging from 1 to 1.5 µm; as an essential feature of hepatocytes, there is a high amount of rough endoplasmic reticulum associated with mitochondria observed in the 5000 × magnification of the control condition (Fig. 2A). Micrographs of HCC group revealed a mitochondria number increased per field (Fig. 2A,B) with decreased mitochondrial length, ranging from 0.3 to 0.7 µm (Fig. 2C). Micrographs of the HCC group showed an increase in endoplasmic reticulum electrodensity and an accumulation of mitochondria within the reticulate spaces. Moreover, the HCC/IFC-305 group recovered mitochondrial length and number at similar levels to the control group, with 1 to 1.5 µm of mitochondrial size and an abundance near the 50 mitochondria per field (Fig. 2B,C). The IFC-305 group shows no significant differences in mitochondrial structure compared to control condition and maintains similar mitochondrial length, abundance, and electrodensity of intracellular organelles. Furthermore, the mtDNA/nDNA ratio (Fig. 2D) showed a significant decrease in the HCC condition compared to the control group; conversely, in the HCC/IFC-305 group, the DNA ratio level was partially recovered. Finally, the IFC-305 group showed no change in the mtDNA/nDNA ratio in comparison with the control group.

### The administration of IFC-305 induces an increase of mitochondrial phospholipids

**Figure 3:**
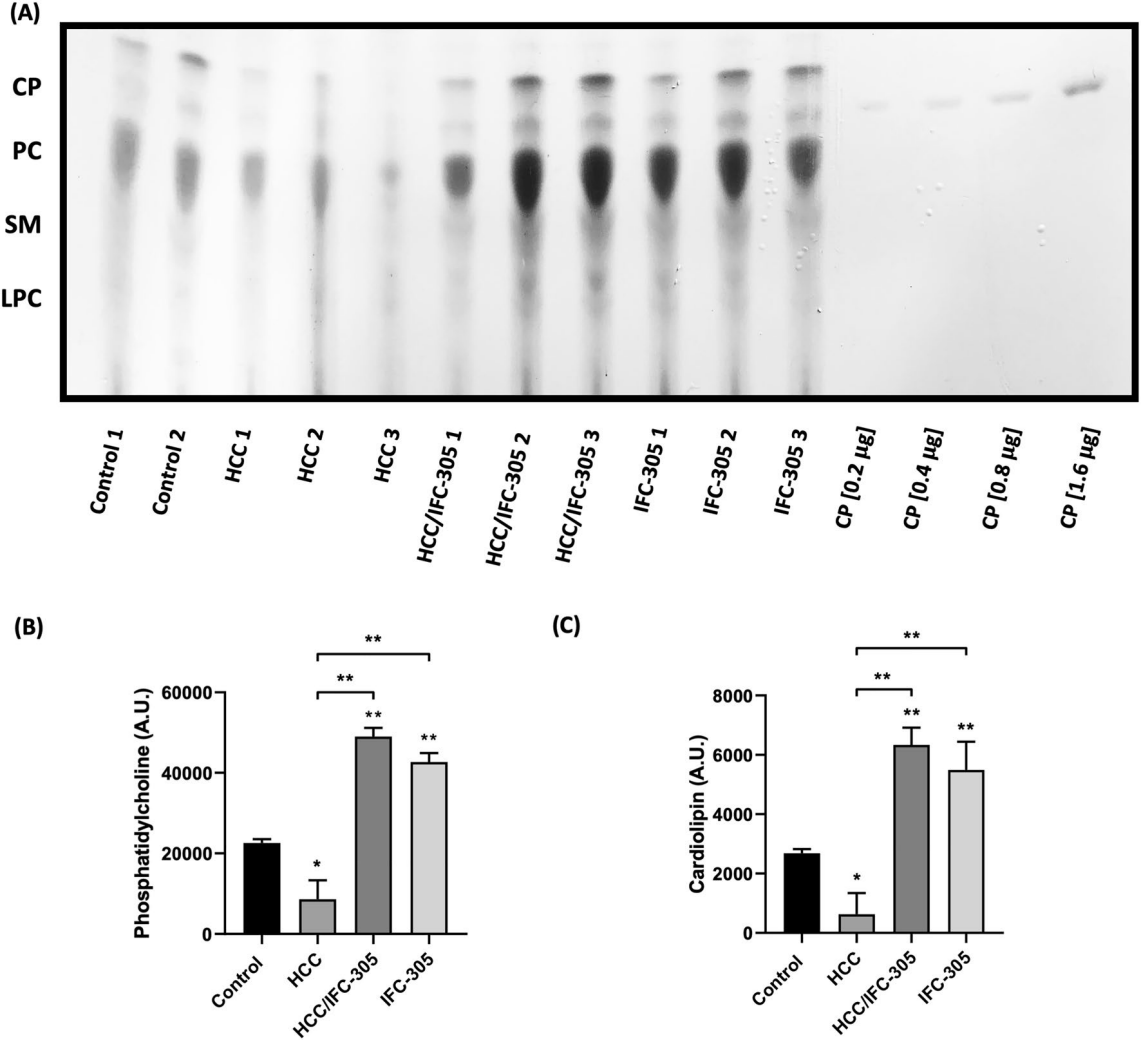
Determination of mitochondrial phospholipids. (**A**) Thin layer chromatography with the abundance of phospholipids in representative samples of the experimental groups: Cardiolipin (CP), phosphatidylcholine (PC), sphingomyelin (SM), lysophosphatidylcholine (LPC); (**B**) PC and (**C**) CP levels expressed in arbitrary units (A.U.) are graphically represented (n = 5 per group). Data are means ± SEM; Kruskal–Wallis with Dunn’s test as post hoc * *P* < 0.05, ** *P* < 0.01, and *** *P* < 0.001.

Phospholipids play a critical role in the structure of mitochondria and assembly of mitochondrial complexes^23^; for this reason, we evaluated the mitochondrial cardiolipin and phosphatidylcholine levels. Figure 3A represents the total phospholipid abundance in two control and three samples of each treatment scheme. When comparing the phospholipids levels in each sample, there is a decrease in cardiolipin (CP) and phosphatidylcholine (PC) levels in HCC samples compared to control condition (Fig. 3A–C). Moreover, HCC/IFC-305 group showed increased levels of CP and PC compared to HCC and control groups. Interestingly, the administration of IFC-305 in healthy rats increased the phospholipid levels compared to the HCC and control conditions (Fig. 3A–C).

### IFC-305 promotes the mitochondrial hmwSC assembly

**Figure 4:**
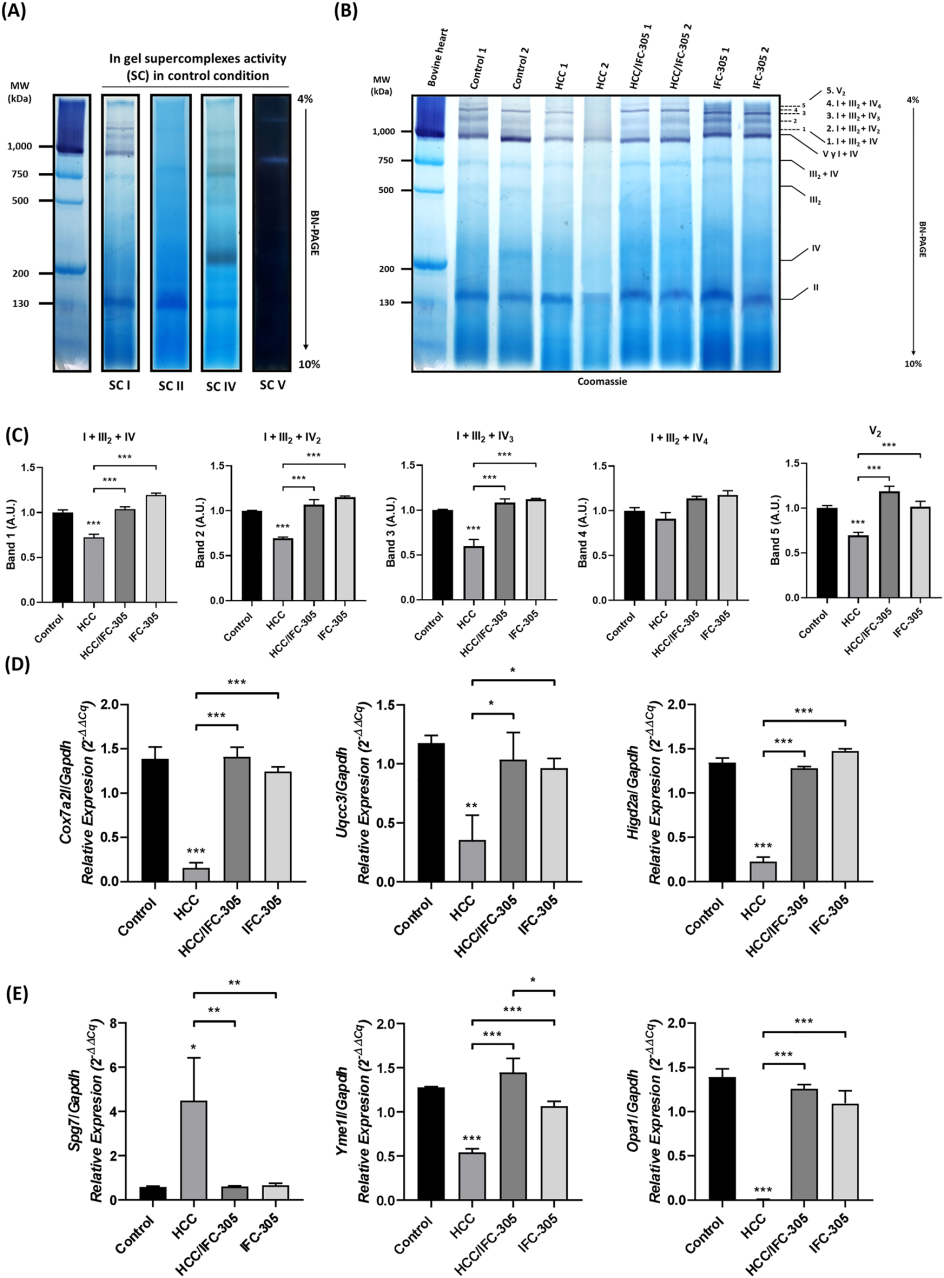
Determination of mitochondrial supercomplexes and involved genes. (**A**) The activity of supercomplexes I, II, IV, and V in polyacrylamide blue native gels (BN-PAGE). The samples were previously treated with digitonin for preserves the interactions between SC so that the monomer and the low and high molecular weight SC are observed in their corresponding weight (**B**) Mitochondrial supercomplexes were identified in the different experimental groups by complex I activity and Coomassie blue staining in BN-PAGE. The first lane corresponds to bovine heart mitochondria previously solubilized with lauryl maltoside, a detergent that does not preserve interactions between SCs, and, therefore, it can be used to observe mitochondrial complexes in their monomeric form. (**C**) Densitometric analysis of the supercomplex bands (n = 5 per group). (**D**) Determination of mRNA expression levels by RT-qPCR by the comparative Ct method for *Cox7a2l*, *Uqcc3*, *Higd2a,* and (**E**) *Spg7*, *Yme1l,* and *Opa1* genes (n = 5 per group). Data are means ± SEM of the five animals per group analyzed by triplicate. One-way ANOVA with Bonferroni test as post hoc *P < 0.05, ** *P* < 0.01, and *** *P* < 0.001.

There is a close relationship between CP levels and mitochondrial hmwSC assembly^20^; hence, hmwSC were determined in the different experimental groups. In Fig. 4A, the panel shows the activity of complexes I, II, IV, and V and, the Fig. 4B, the mitochondrial hmwSC are identified by BN-PAGE in the different treatment schemes as described in “Methods” section; the first lane corresponds to bovine heart mitochondria and this sample was used as a molecular weight marker since the bands corresponding to each mitochondrial complex are widely reported previously^24^. In the other lanes, the activity bands corresponding to supercomplex I (hmwSC I) of the experimental group samples are shown to identify bands in which mitochondrial hmwSC are positioned compared with the activity of hmwSC II, IV, and V (Fig. 4B). Our results exhibited that in the control condition, 5 hmwSC were identified (Fig. 4B labels 1–5 and 4C). In contrast, the HCC group shows a decrease in hmwSC, particularly hmwSC 1, 2, 3, and 5, identified as I + III_2_ + IV, I + III_2_ + IV_2_, I + III_2_ + IV_3_, and V_2_. Interestingly, HCC/IFC-305 group showed a recovery of hmwSC identified as 1, 2, 3, and 5 (Fig. 4B,C). Finally, when comparing the IFC-305 group with the other experimental conditions, it is observed that all the identified hmwSC are present and their levels are similar to control group (Fig. 4B,C). In addition, the expression levels of mRNAs that codified the respirosome assembly (*Cox7a2l*, *Uqcc3,* and *Higd2a*) (Fig. 4D) and the structural maintenance of the mitochondrial cristae (*Opa1* and indirectly *Yme1l* and *Spg7*) were determined (Fig. 4E); the results showed a decrease in the expression levels of *Cox7a2l*, *Uqcc3,* and *Higd2a* in the HCC condition compared to the control group; nevertheless, the expression of these evaluated mRNAs is similar to the control group in the HCC/IFC-305 one. Additionally, no changes concerning the control condition were observed in the IFC-305 group. Conversely, the *Yme1l* expression levels are down-regulated in the HCC condition, but *Spg7* is up-regulated in this experimental group. Finally, the expression levels of *Opa1* are down-regulated in the HCC condition and were recovered with the IFC-305 treatment; no significant changes were observed in the group treated only with IFC-305 (Fig. 4E).

### The structural modifications promoted by IFC-305 improve mitochondrial function

Coupling between the electron transport chain and ATP production by OXPHOS indicates proper mitochondrial function. Therefore, metabolic aspects were determined, such as the amount of ATP, where it is shown that the HCC group exhibited a decrease in the liver ATP levels, while it increased significantly when IFC-305 was administered (Table 1). Additionally, we measured ψm, which demonstrated a depolarization of ψm in the HCC group and its recovery when the adenosine derivative is administered, indicating that directly affects the mitochondrial homeostasis (Table 1). To evaluate the efficiency of ETC, the activity of the mitochondrial complexes was measured, and the results showed a decrease in the activity of complex I in HCC group concerning the control; nevertheless, the activity increased with a significant statistical difference in HCC/IFC-305 and IFC-305 groups compared to HCC condition. Interestingly, in IFC-305 group, there is a considerable increase in the activity of this complex. In contrast, in succinate dehydrogenase activity (complex II), there is a tendency to decrease in the HCC group compared with the control group. Still, there is no significant difference between the four experimental groups. However, when evaluating the activity of cytochrome bc1 (Complex III), a decrease in HCC group activity is observed, and this activity recovers when IFC-305 is administered (Table 1).

**Table 1:** Assessment of mitochondrial function. Measurement of factors determining mitochondrial status, amount of ATP in tissue, mitochondrial membrane potential, and complex I, II (By DCPIP), and III (By Enzymatic assay of cytochrome c reductase method) activity (n = 5 per group). Data are represented as means ± SEM. One way ANOVA and Bonferroni test as post hoc ^**a**^ *P* < 0.05 difference concerning the control; ^**b**^ *P* < 0.05 difference between the HCC/IFC-305 group concerning HCC condition. ^**c**^ *P* < 0.05 difference between IFC-305 in contrast to the HCC group.

	ATP [pmol/mg] ± SEM	Mitochondrial membrane potential [mV] ± SEM	Complex I Activity [μmol/min*mg] ± SEM	Complex II Activity [μmol/min*mg] ± SEM	Complex III Activity [mU/mg] ± SEM
Control	38.6 ± 11.14	-148 ± 7.06	5.837 ± 1.19	0.629 ± 0.08	0.253 ± 0.04
HCC	22.38 ± 3.54 ^a^	-78.4 ± 16.70 ^**a**^	2.757 ± 0.35 ^**a**^	0.427 ± 0.09	0.099 ± 0.02 ^**a**^
HCC/IFC-305	57.94 ± 7.32 ^**b**^	-146.4 ± 19.82 ^**b**^	6.063 ± 0.99 ^**b**^	0.766 ± 0.07	0.247 ± 0.08
IFC-305	36.32 ± 6.54	- 175.6 ± 17.55 ^**c**^	23.88 ± 7.70 ^**c**^	0.564 ± 0.10	0.301 ± 0.04 ^c^

### Role of IFC-305 in the regulation of genes involved in mitochondrial bioenergetics

**Figure 5:**
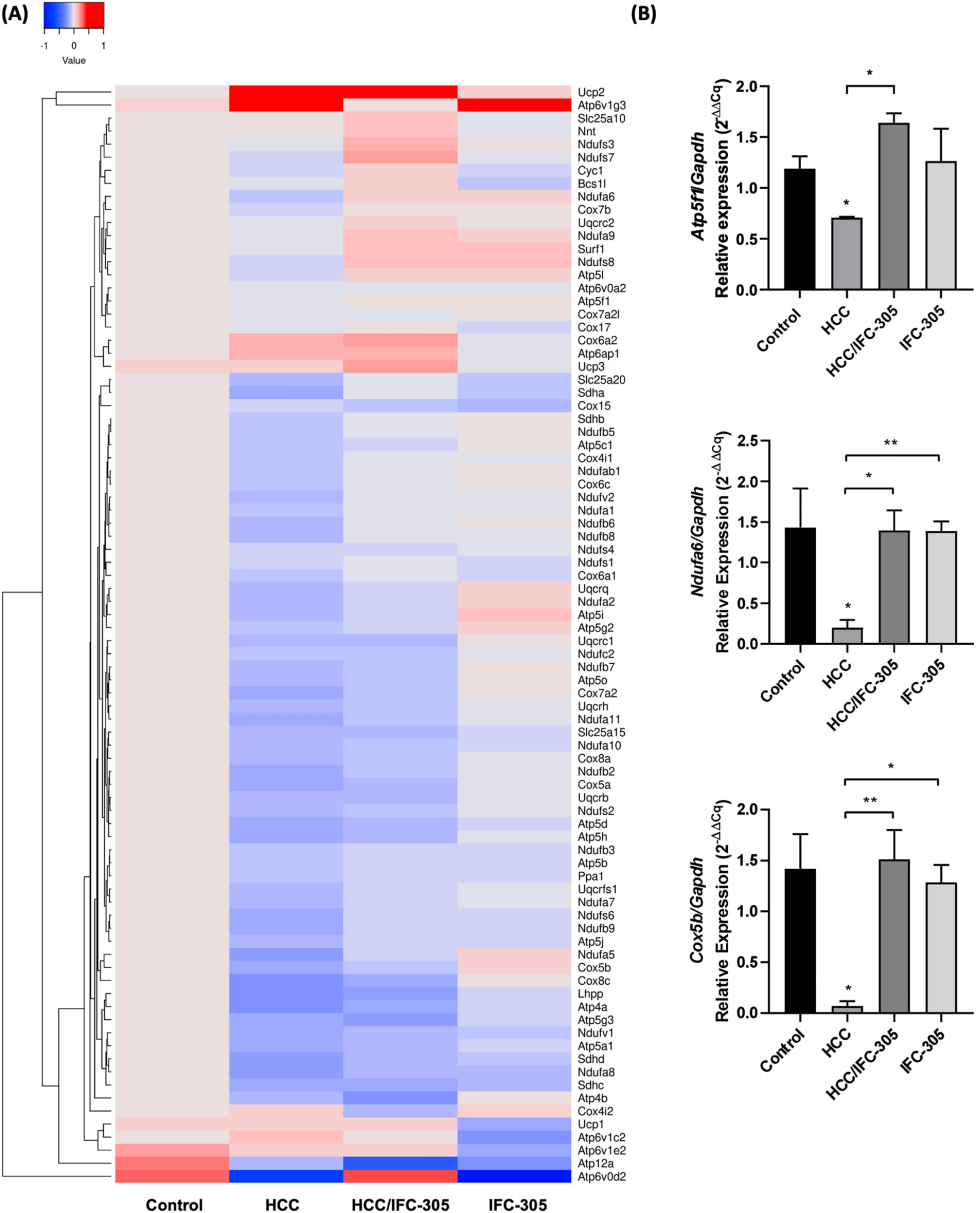
Evaluation of genes related to mitochondrial bioenergetics. Expression profile of mitochondrial energy metabolism genes with the QIAGEN microarray from liver tissue total RNA. (**A**) Microarray data (Ct) obtained from qPCR was normalized to the *Gapdh* reference gene (ΔCt) and represented as the relative expression (2^-ΔΔCq^) concerning the control group in the log scale (n = 3 per group). (**B**) mRNA expression levels of *Atp5f1* from complex V, *Ndufa6* from complex I, and *Cox5b* from complex IV. Data (Ct) obtained from qPCR are normalized to the reference gene *Gapdh* (∆Ct) and normalized to relative expression (2^-∆∆Ct^) concerning the control group. Data are means ± SEM of the five animals per group analyzed by triplicate. One-way ANOVA and Bonferroni test as post hoc *P < 0.05, ** *P* < 0.01, and *** *P* < 0.001.

To identify the elements that modulate the efficiency in mitochondrial bioenergetics and participation of IFC-305 in this biological process, a 84 genes microarray of mitochondrial bioenergetics was performed, which contained oligonucleotides for determining the expression levels of relevant subunits in each complex of the ETC and accessory proteins (Fig. 5A). The results show a differential expression pattern between the experimental groups; in DEN group, most genes encode elements that compose complexes I, II, III, IV, and V of the mitochondrial ETC are down-regulated. In contrast, the genes *Ucp2* and *Ucp3* (Proton carrier/OXPHOS uncoupler), *Cox6a2* and *Cox4i2* (complex IV), *Atp6ap1*, *Atp6v1g3* and *Atp6v1c2* (ATPase H^+^ transporting lysosomal) are up-regulated. In addition, the expression of some elements of the different complexes recovers the expression level in the HCC group with IFC-305 to similar levels of the control group, including *Slc25a20* (Solute carrier family), *Uqcrfs1* (Complex III), *Ndufb5*, *Ndufab1*, *Ndufs6*, *Ndufv2*, *Ndufa1*, *Ndufb6*, *Ndufb8*, *Ndufs1*, *Ndufb9*, *Ndufb3*, *Ndufa5* and *Ndufa7* (Complex I), *Sdha* and *Sdhb* (Complex II), *Cox4i1*, *Cox6c* and *Cox6a1* (Complex IV), *Ppa1* (Inorganic diphosphatase), *Atp5b*, *Atp5f1* and *Atp5j* (Complex V). Nevertheless, the genes *Ucp2* and *Ucp3*, *Slc25a10* (Solute carrier family), *NnT* (NAP (P) transhydrogenase)*, Surf1* (Complex IV), *Ndufs3*, *Ndufs7*, *Ndufa6*, *Ndufa9* and *Ndufs8* (complex I), *Cyc1*, *Bcs1l* and *Uqcrc2* (complex III), *Cox6a2* (complex IV), *Atp5l* (Complex V) and *Atp6ap1* (ATPase H^+^ transporting lysosomal), are up-regulated. The experimental group IFC-305 shows genes that remain with similar expression to the control; however, these elements have a down-regulated expression, *Slc25a20*, *Ucp1* (Proton carrier/OXPHOS uncoupler), *Ndufab1* and *Ndufa8* (complex I), *Sdha*, *Sdhd* and *Sdhc* (complex II), *Cox15* (Complex IV), *Atp4a and Atp12a* (ATPase H^+^/K^+^ exchanging), *Atp5g3* and *Atp5a1 (Complex V)*, *Atp6v1c2, Atp6v1e2 and Atp6v0d2* (ATPase H^+^ transporting lysosomal). In contrast, *Ucp2*, *Ndufa6*, *Ndufa9*, *Ndufs8*, *Ndufa2* and *Ndufa5* (complex I), *Uqcrq* (complex III), *Cox5b, Surf1* and *Cox4i2* (complex IV), *Atp6v1g3 (*ATPase H^+^ transporting lysosomal), *Atp5l*, *Atp5i* and *Atp5g2* (complex V) are up-regulated. These results indicate that the IFC-305 compound may influence the modulation of mitochondrial complexes and energy production, thereby intervening in the recovery of mitochondrial functions that are impaired in HCC.

Some relevant genes were determined to validate the differential expression of components of the ETC (Fig. 5B). First, the *Atp5f1* gene was analyzed, which encodes the b subunit of the peripheral stalk of ATP synthase; the results exhibited a down-regulation in the HCC group with a recovery in HCC/IFC-305 group to similar levels compared with the control group. Additionally, *Ndufa6*, a gene that encodes an essential protein for complex I function, was analyzed. The validation of this gene shows a decrease in expression in HCC, which is recovered when the compound IFC-305 is administered. Finally, the relative expression levels of the *Cox5b* gene, which encodes to 5b subunit of cytochrome C oxidase, were measured. The results showed a down-regulation in the HCC group with a marked recovery in the HCC/IFC-305 condition to similar expression levels to the control group. Therefore, these results confirm the microarray tendency and validate the expression profile presented by the Heatmap.

### IFC-305 restores ETC complexes activity in HCC

**Figure 6:**
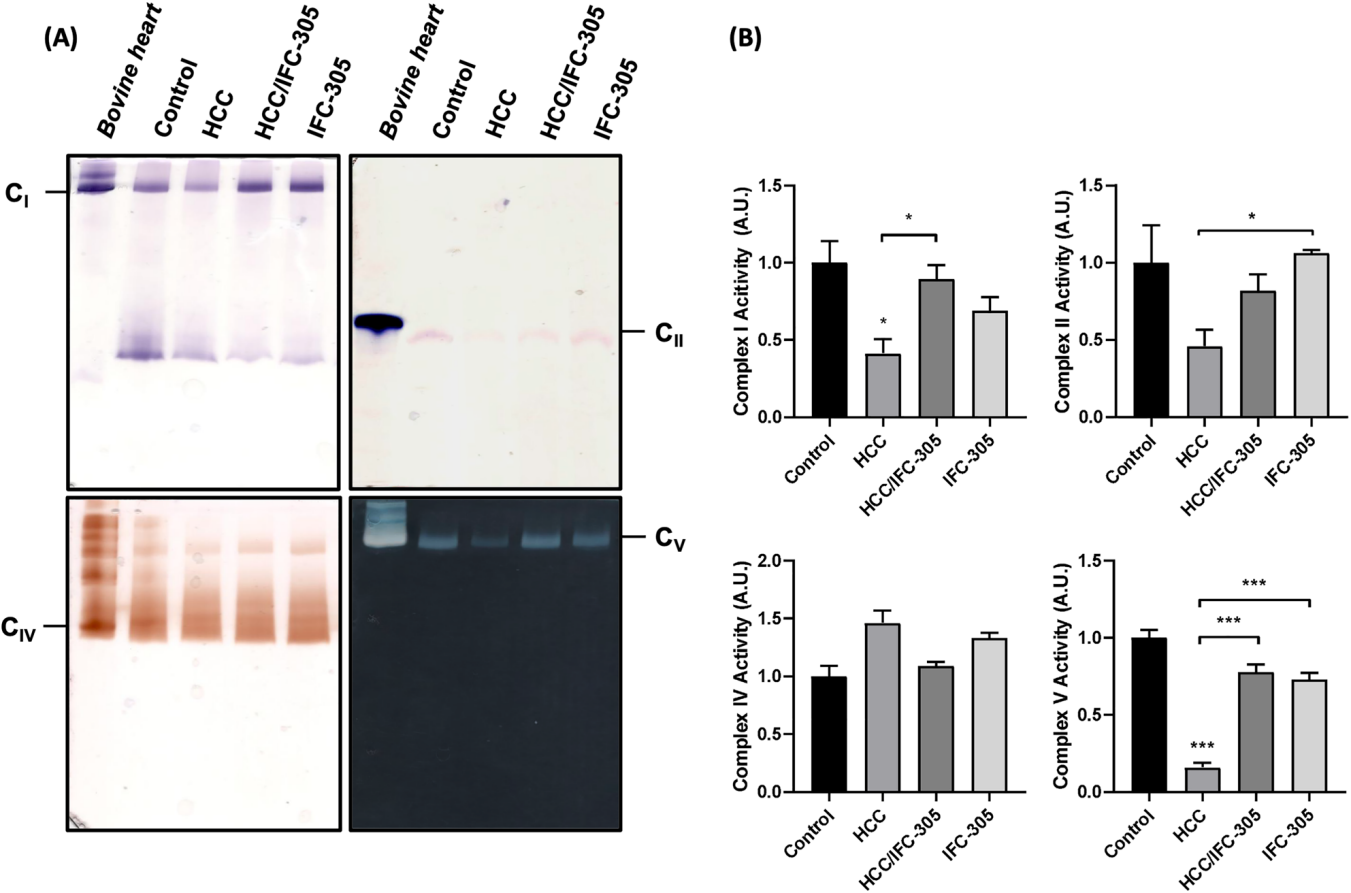
Activity of Complex I, II, IV, and V. (**A**) Clear Native Polyacrylamide Gel Electrophoresis (CN-PAGE) representative of the activity of mitochondrial complexes I, II, IV, and V using rat liver mitochondria from the control group, HCC, HCC/IFC-305, and IFC-305. Bovine heart mitochondria were used as a positive control to determine the location of the bands shown in the gels. (**B**) Densitometric analyses of the bands obtained in CN-PAGE of their respective mitochondrial complex activity. Data are means ± SEM of the five animals per group analyzed by triplicate. One-way ANOVA with Bonferroni test as post hoc *P < 0.05, ** *P* < 0.01, and *** *P* < 0.001.

Measurement of enzymatic activity of NADH:ubiquinone oxidoreductase complex (CI), succinate dehydrogenase (CII), cytochrome c oxidase (CIV), and substrate-mediated ATP synthase (CV) was performed using the clear native polyacrylamide gels (CN-PAGE) technique (Fig. 6A,B) adding the same amount of mitochondrial protein of each condition in each gel. The NBT method was implemented to detect complex I and NADH alternating dehydrogenases, the results showed a decrease in the activity of complex I in the HCC group concerning the control group. Nevertheless, upon administration IFC-305, the complex activity showed no significant difference compared to the control group. Densitometry graphs displayed a statistically significant difference between the control and HCC groups and between the HCC and HCC/IFC-305 groups, indicating that the compound can functionally restores one of the most critical complexes in OXPHOS. The same method was implemented in complex I, changing NADH for succinate dehydrogenase; the results indicated a decrease in activity by the HCC group and recovery when administering the IFC-305 compound; however, when performing the densitometric analysis, there is only a significant statistical difference between HCC and IFC-305 groups. The results of the measurement of cytochrome c oxidase to detect the activity of complex IV using 3’, 5’-diaminobenzidine, and horse heart cytochrome c, showed that the activity remains similar in all groups, there are no statistical differences, corroborating that the activity of this complex is not modified in HCC or by IFC-305 administration. Finally, the reaction of lead nitrate with a phosphate buffer was necessary for forming lead phosphate and identifying complex V activity. The results showed a very low ATPase activity in the HCC group; however, after administering IFC-305, there was a significant recovery in the complex activity. The gel densitometry analysis determined a statistically significant difference between HCC and control groups in complex I and V activity; HCC/IFC-305 in complex I activity; IFC-305 in complex II activity, and HCC/IFC-305 and IFC-305 in complex V activity.

## Discussion

Mitochondrial dysfunction is one of the main features of HCC progression^25^. These cytoplasmic organelles play multiple roles in energy metabolism and cellular homeostasis, including the generation of ATP via respiration and OXPHOS, ROS production, metabolic homeostasis, and apoptosis^26^. Mitochondrial dysfunction has been linked to several degenerative and metabolic diseases, aging, and cancer^27^. IFC-305 is a compound with potential for HCC treatment with effect on mitochondrial dynamics. Therefore, we aimed to determine the IFC-305 effect on the mitochondrial structure and bioenergetics in a sequential cirrhosis-HCC model. Firstly, the molecular and biochemical markers for liver damage, proliferation, and fibrosis were analyzed for HCC model validation; the expression levels of *Afp* and *Gpc3* provide high specificity for HCC detection^28^. In addition, *Mki67* indicates a high proliferative rate^29^ and *Col1a1* the fibrosis level in tumoral liver tissue^30^. The elevated mRNA expression levels of the molecular markers validate that the rats with hepatic tumors corresponded to HCC. In contrast, a decrease in the expression levels of these mRNAs, the restoration of liver tissue architecture, and the reduction of AFP serum levels in the HCC/IFC-305 group indicated the effectiveness of IFC-305 in reversing HCC.

The TEM results showed that under normal conditions, the mitochondria in the liver tissue of rats have a morphology with predominantly elongated structures; this feature corresponds to a dynamic mitochondrial process involving fusion and fission of the structure as part of quality control to preserve and maintain active mitochondrial function^16^. Conversely, under a loss of this mitochondrial quality control, the mitochondrial fusion and fission rate can be altered; according to the results, in HCC group, there is a decrease in the mitochondrial size that coincides with previous studies where a blockage of mitophagic flow is demonstrated in liver tissue in the same experimental model used here^22^; preventing mitophagy from being carried out entirely generates a decrease in the damaged mitochondria degradation, causing an abnormal accumulation of fissioned, smaller and non-functional mitochondria^5^. In HCC, it has been reported the overexpression of PGC-1α^5,31^, one of the regulators of mitochondrial biogenesis that activates several transcription factors such as nuclear respiration factor 1 and 2 (NRF-1 and NRF-2, respectively), promoting the transcription of elements required for mitochondrial biogenesis^32^. In contrast, it has been demonstrated that IFC-305 restores the protein levels of PGC-1α in an HCC and cancer progression model to levels similar to the non-tumoral condition^5^.

Mitochondria in the HCC group showed high electrodensity in the endoplasmic reticulum; this could be generated by misfolded protein accumulation in the reticular lumen and, therefore, generating areas of higher density with higher electron absorbance^33^. In the mitochondrial structures corresponding to the HCC/IFC-305 group, a decrease in the electrodensity of the endoplasmic reticulum was observed concerning HCC group, as well as an increase in mitochondrial length in a similar range was observed in the control group, this may imply an improvement in the mitochondrial quality control processes^5^, as well as the regulation of elements not yet described that are related to mitochondrial structure. A decrease in the mtDNA copy number correlates with tumor size, cirrhosis, prognosis, and mitochondrial dysfunction in HCC, given that mtDNA is more susceptible to oxidative damage and has a higher mutation rate relative to nuclear DNA because of the lack of protective histones, limited DNA repair activity, and proximity to sources with high rate of generation of ROS in mitochondria^26,34,35^. Interestingly, when determining the mtDNA/nDNA ratio, we found a decrease in the mitochondrial genetic content, probably because of the mitochondrial damage produced by the pathological condition; this agrees with previous studies that demonstrate a reduction in the mtDNA copy number in HCC in comparison with non-tumoral liver tissue^10^. In contrast, in the HCC/IFC-305 condition, there is a significant recovery in mtDNA levels concerning the HCC condition but not as high to reach the expression observed in the control group. Therefore, these results suggest that IFC-305 modulates mtDNA copy number, which could explain the mitochondrial recovery at the structural and functional level and may participate in liver function restoration.

The generally identified pathways for synthesizing phosphatidylcholine are de novo synthesis, also known as the Kennedy pathway; the Lands cycle, or it can be synthesized by the phosphatidylethanolamine methyl transferase pathway, which is a synthetic pathway exclusive of the liver^36^. Since IFC-305 regulates the levels of S-Adenosylmethionine (SAM: a methyl group donor)^37,38^, it is then inferred that in the groups treated with IFC-305, there is availability of methyl groups required by phosphatidylethanolamine (PE) for PC synthesis; this may explain the increase observed in PC levels in the HCC/IFC-305 and the IFC-305 experimental groups compared to HCC and control conditions. In addition, there is a decrease in cardiolipin levels in the HCC group; the same results have been reported in other types of cancer^38^ and are similar to those obtained here. Cardiolipin plays an essential role in structural aspects by contributing to the formation of curvatures in mitochondrial cristae, bioenergetic aspects, mitophagy, or apoptosis but also has a direct relationship with the formation of mitochondrial hmwSC; thus favoring efficient oxidative metabolism in mitochondria^39^. The amount of cardiolipin in HCC decreases, which coincides with the alterations we found in mitochondrial structure and bioenergetics as well as in genes related to cristae maintenance, as we describe in the “Results” section. Moreover, cardiolipin levels are recovered in the HCC/IFC-305 group, suggesting that IFC-305 might impact mitochondrial structure recovery as observed in TEM micrographs. Due to the low cardiolipin levels in the HCC group and considering its relationship with the formation of mitochondrial hmwSC, we expected mitochondrial alterations of hmwSC in the experimental groups. Since hmwSC are dynamic structures in the mitochondrial inner membrane, they aggregate in different stoichiometric combinations to form respirosomes and perform adequate cellular respiration^40^. Our results suggest that not only is the composition of structural elements modified but also that structural alterations have functional consequences at the mitochondrial level. Furthermore, we evaluated the expression of genes encoded to complex coupling proteins COX7A2L, UQCC3, and HIGD2A and the mitochondrial dynamin-like GTPase OPA1 and their regulatory proteases. Our results explain that the formation of hmwSC is consistent with the expression levels of hmwSC assembly-related genes. Besides, paraplegin and YME1L employ OPA1 as substrate; the increased expression levels of paraplegin could explain the observed decrease in OPA1 expression^20^, supporting the integrity of the mitochondrial cristae structure and preserving the mitochondrial function^5,21^. We suggest that the down-regulated mRNA *Opa1* and up-regulated *Spg7* expression levels in HCC are fundamental events for induction of mitochondrial alterations.

Complexes I, III, IV, and V activities are not modified in the Myc-driven HCC model^41^. Nevertheless, we determined that in the HCC DEN-induced model, the ATP production decreases, as well as ψm and the activity of complexes I and III showed a significant reduction; it has been demonstrated that complex I is mainly affected by the DEN agent and complex III by the HCC condition^12,13^. Previous reports suggested mitochondrial damage in HCC and the participation of IFC-305 in recovery^5^. However, this is the first evidence to associate changes in mitochondrial structure with the beneficial effects of the compound IFC-305.

The expression levels of genes related to the activity of mitochondrial complexes I, II, III, IV, and V are differentially expressed under the HCC condition and modulated by IFC-305 administration. Therefore, we analyzed some crucial genes related to mitochondrial bioenergetics; first, the *Atp5f1* gene was analyzed, the b subunit of the peripheral stalk that links the external surface of the catalytic domain (F_1_) to the membranal domain (F_0_) of ATP synthase^42^. Additionally, *Ndufa6* was analyzed, a gene that encodes a protein with a conserved tripeptide sequence near the N-terminus that is important for complex I function; its deficiency is associated with decreased complex I activity and mitochondrial diseases^43^. Finally, we also analyzed the relative expression levels of the *Cox5b* gene, the 5b subunit of cytochrome C oxidase, which is involved in assembling complex IV and its proper function^44^. These genes maintained a differential expression between groups; therefore, it is concluded that the reduced expression of these genes in HCC and the recovery in the other conditions suggest that IFC-305 regulates nuclear and mitochondrial encoded genes related to mitochondrial bioenergetics. These observations were confirmed with the increased ATP levels by recovering the complex I, III, and V activity.

Unlike systemic drugs therapies for HCC clinically available, such as multi-kinase inhibitors (Sorafenib, lenvatinib, cabozantinib, regorafenib), antagonistic anti-VEGFR2 monoclonal antibody (ramucirumab), immune checkpoint inhibitors (nivolumab and pembrolizumab) and combined alternatives which induce hepatocytes apoptosis^45^, or pre-clinically in vivo or in vitro tested compounds that decrease the mitochondrial function in HCC such as biguanide drugs (metformin and phenformin)^46^, sulfonamides FH535 and Y3^47^ and sorafenib with nitazoxanide or nidosamide^48^. The chemoprotective effect of IFC-305 was demonstrated by the modulation of the expression PCNA, thymidylate synthase, HGF, and its receptor c-Met and p27^3,4^. As part of its mechanism of action, its effect on the recovery of mitochondrial homeostasis through the induction of autophagy has been proposed. In this work, our results suggest that IFC-305 restores the mitochondrial structure and function in liver tissue.

**Figure 7:**
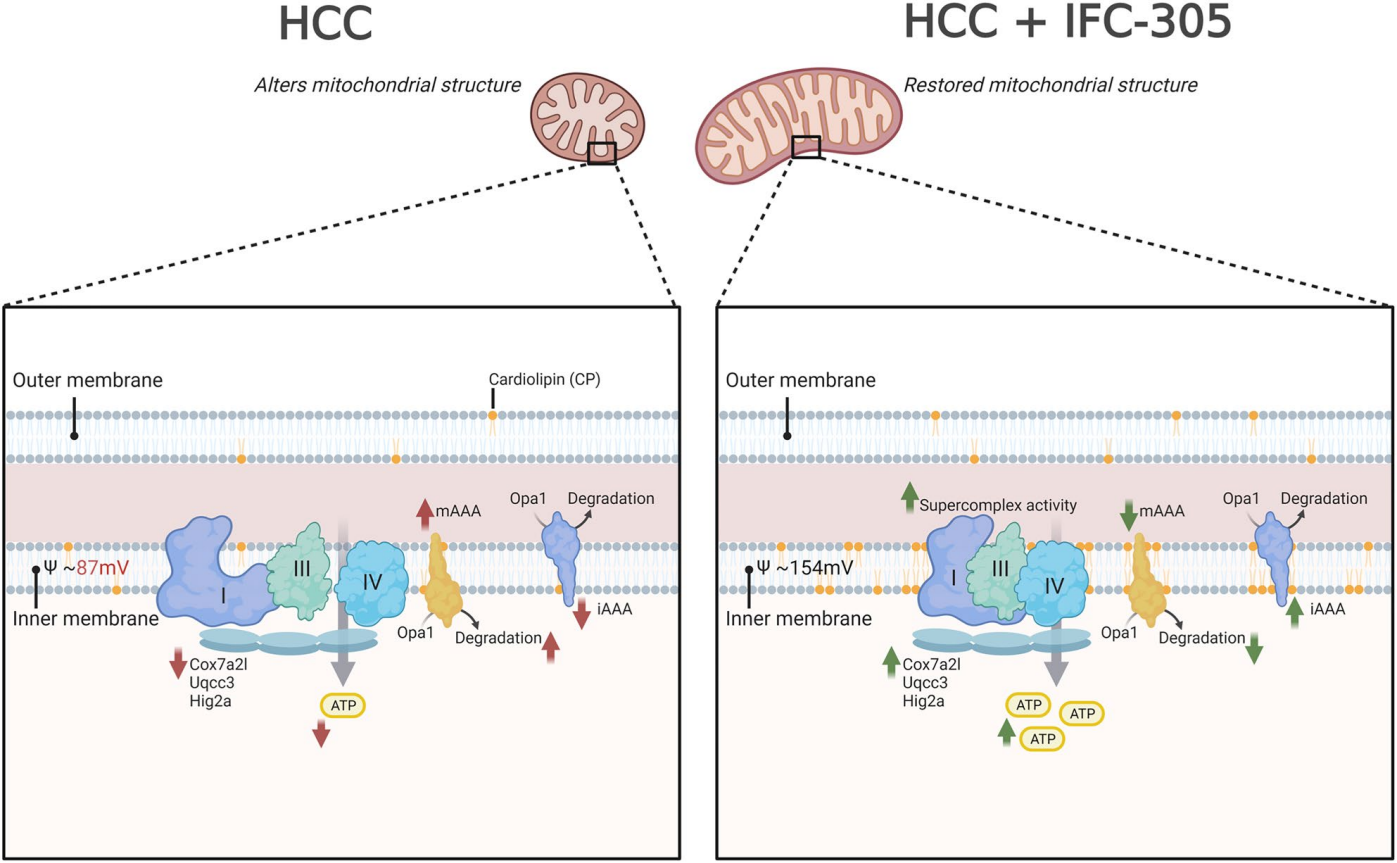
Proposed mechanism of mitochondrial structural and functional changes by IFC-305 in HCC. The left side of the panel shows the mitochondrial structural and functional changes in HCC; it represents an uncoupling of the electron transport chain complexes as well as a decrease in mitochondrial length, respiration rate, ψm, cardiolipin levels, ATP concentration, and the levels of YME1L, OPA1, COX7A2L, UQCC3, HIGD2, and increased expression of paraplegin. On the right side of the panel are shown the changes that occurred in the HCC condition treated with IFC-305, representing substantial changes in the formation of the respirosome (SC I, II_2_, IV_n_), a recovery in mitochondrial length, respiration rate, ψm, cardiolipin levels, mitochondrial complex activity, ATP levels, and expression levels of YME1L, OPA1, Paraplegin, COX7A2L, UQCC3, and HIGD2A. The figure was created with BioRender.com with agreement number NM25VM5FTL.

Finally, our findings show an association between mitochondrial structure and function, and the IFC-305 impacts both aspects. In HCC (Fig. 7 left panel), it has been previously suggested that a change in mitochondrial structure coupled with an increased fission rate allows the survival of liver tumor cells by a poorly elucidated mechanism^49^, just as shown in our results by observing an increase in the number of mitochondria in the HCC condition. Furthermore, accompanied by the rise in fission rate, there are structural and functional changes in mitochondria leading to metabolic alterations^6^ that involve changes in ψm, ATP production, the activity of ETC complexes^5^, as well as a decrease in cardiolipin levels^50^. The supercomplexes formation conditions have been poorly documented until now. Concurrently, the participation of structures related to the assembly of supercomplexes, such as COX7A2L, UQCC3, and HIGD2A, whose relevance lies in the correct function of the respirosome formed by complexes I, III_2_, and IV_n_, has been reported^20^; our data supported these conclusions since we found a considerable decrease in expression levels of mRNAs in the HCC condition, which correlates with the structural (formation of supercomplexes) and functional (ATP production) nature. On the other hand, the right scheme of Fig. 7 shows the activity of IFC-305 on several structural and functional aspects that allow the functional recovery of mitochondria, possibly as part of the liver restoration effect induced by the compound. Nevertheless, the precise mechanism by which IFC-305 modulates the structural components of mitochondria and their function remains unknown; however, it is a topic of interest for future research.

## Materials and methods

### Chemicals

IFC-305 is the aspartate salt of adenosine prepared with adenosine-free base (MP Biomedicals, LLC, Illkirch, France) and l-aspartic acid (MP Biomedicals, Inc., Eschwege, Germany) as described (Patent No. MX220780; MX 207,422; US 8,507,459 B2). Diethylnitrosamine, sucrose, EDTA, trizma-base, KCl, MgCl_2_, glutamate, ADP, succinate, 2,6-dicholorindophenol, ATP, NAD, NADH, lauryl-maltoside, cytochrome c, albumin, deoxycholate, nitrotetrazolium blue chloride (NBT), ɛ-aminocaproic acid, acrylamide, malate, potassium cyanide (KCN), antimycin A, oligomycin, rotenone, methanol, chloroform, Coomassie blue and digitonin were purchased from the Sigma Chemical Company (St. Louis, MO, USA). The oligonucleotides were manufactured by the OligoT4 Company (Irapuato, Guanajuato, Mexico).

### Animal model and ethical statement

Male Wistar rats weighing 250 to 300 g were housed at the Animal Facility of the Universidad Nacional Autónoma de México (National authorization SAGARPA-SENASICA AUT-B-C-1216–030). All animal experiments were conducted in compliance with the Mexican federal regulations NOM-ZOO-1999-062 regarding the protection of animals used for scientific purposes and received approval by the Ethics Committee for the Care and Handling of Laboratory Animals of the Universidad Nacional Autónoma de México with national authorization SAGARPA-SENASICA AUT-B-C-1216-030 (permission number of institutional protocol approval CICUAL-VCHH156-19). All procedures were approved and supervised, and this study was reported in accordance with the ARRIVE guidelines. The animals were fed ad libitum and housed under controlled conditions (22 ± 2 °C, 50–60% relative humidity, and 12 h light–dark cycles). The rats were divided in four groups: the control group (C), hepatocellular carcinoma (HCC), HCC/IFC-305 group, and IFC-305 group. HCC and HCC/IFC-305 received DEN (50 mg/kg body weight, i.p. for 16 weeks once a week), while the IFC-305 group received saline solution i.p. for 16 weeks once a week. Subsequently, HCC received a saline solution, and the IFC-305 and HCC/IFC-305 groups were administered with IFC-305 at 50 mg/kg body weight, i.p. five times a week for 6 weeks starting at week 16. The control group received saline solution for 22 weeks. The animals were anesthetized by pentobarbital administration, the blood was collected by cardiac puncture, and the liver was removed for future determinations.

### ALT levels determination in liver tissue

The Alanine aminotransferase (ALT) levels were determined using the ALT Activity Assay (Colorimetric/Fluorometric) (Cat. MAK052 Sigma-Aldrich). The instructions provided by the manufacturers were followed.

### GGT levels determination in liver tissue

According to the manufacturer instructions, the γ-glutamyltransferase (GGT) levels were determined using the γ-Glutamyltransferase Activity Fluorometric Assay Kit (Cat. MAK090 Sigma-Aldrich).

### AFP levels determination in serum

The alpha-fetoprotein (AFP) levels were determined using the Rat Alpha-Fetoprotein ELISA Kit (Cat. RK03475 ABclonal) following the manufacturer instructions.

### Mitochondria isolation

After sacrifice, 1 g of liver tissue was washed with STBE medium (250 mM sucrose, 10 mM trizma base, and 1 mM EDTA) with 0. 4% albumin (BSA) and then homogenized in 6 mL of STBE medium employing a sterile teflon homogenizer; the solution was then centrifuged at 3000 rpm for 5 min at 4 °C, and 5 mL of the supernatant were taken and further centrifuged at 10,000 rpm for 10 min at 4 °C. The sediment (mitochondria) was washed once with 1.5 mL of STBE medium and centrifuged at 10,000 rpm for 10 min at 4 °C. Finally, the supernatant was removed and resuspended with 700 μL of STBE medium.

### RNA extraction

25 to 30 mg of liver tissue from all groups were processed with the Aurum Total RNA kit (Biorad No. 732-6820). First, the tissue was homogenized with 700 µL of lysis buffer (guanidinium thiocyanate and β-mercaptoethanol) and transferred to a 1.5 mL solvent-resistant conical microtube. The sample was centrifuged at 13,000 rpm for 5 min at room temperature. Subsequently, the supernatant was transferred to a new tube, and 700 µL of 70% ethanol was added and homogenized. Next, 700 µL of the mixture was transferred to an RNA affinity column and centrifuged at 13,000 rpm. After centrifugation, 700 µL of low stringency buffer was added to the column and then centrifuged at 13,000 rpm. Then, 80 µL of DNase was added to the column, and the sample was incubated for 15 min at room temperature. After incubation, a second wash was performed with 650 µL of high stringency buffer, centrifuged at 13,000 rpm. Finally, a last wash was performed with 700 µL of low stringency buffer and centrifuged at 13,000 rpm, followed by dry centrifugation at 13,000 rpm. To perform the elution, 20 µL of water free of RNAsas was used. Subsequently, the RNA concentration was quantified using the Quanti Fluor RNA System kit in a Quantus fluorometer (Promega). Finally, the RNA quality was analyzed in a QIAxpert high-speed UV/VIS microfluidic spectrophotometer (Qiagen).

### Quantitative reverse transcription-polymerase chain reaction (RT-qPCR)

The reverse transcription was performed with 0.5 μg of RNA in the iScript Synthesis kit (Biorad No. 170-8891) for 5 min at 25 °C, 30 min at 42 °C and 5 min at 85 °C to obtain cDNA; then, it was mixed with the corresponding oligonucleotides (the designs were realized using primer3 and primer-BLAST from NCBI, and the qualities were evaluated by OligoAnalyzer Tool from IDT, OligoEvaluator from Sigma-Aldrich, uMelt, and Multiple Primer Analyzer from Thermo Fisher Scientific; Supplementary Table 1) with iQ SYBR Green (Bio-Rad) to perform real-time quantitative PCR (RT-qPCR) on a CFX96 thermal cycler (Bio-Rad) with the following parameters: 3 min at 95 °C and 40 amplification cycles (15 s at 95 °C and 60 s at 60 °C), followed by a melting curves analysis using a temperature from 55 to 95 °C with an increment of 0. 5 °C every 2–5 s/cycle. Data from qPCRs were obtained using the CFX manager program (Biorad) and processed using the comparative Ct method^51^. Each sample was analyzed in triplicate and normalized with the levels of the reference gene (ΔCt) and compared against the experimental conditions considered as control (ΔΔCt) to subsequently obtain the relative expression (2^-ΔΔCt^) as the mRNA expression level compared to the control condition.

### DNA extraction and mtDNA/nDNA ratio

A 10 mg of liver tissue sample was processed from all groups with the QIAmp DNA Micro Kit (QIAGEN No. 56304) following the manufacturer instructions. As described above, 600 ng of DNA obtained were mixed with the corresponding oligonucleotides (supplementary Table 1) with iQ SYBR Green (Bio-Rad) and parameters as previously described. Data from qPCRs were obtained using the CFX manager program (Biorad) and processed using the comparative Ct method^51^. The mitochondrial gene *Cox1* was analyzed in triplicate and normalized with the levels of the nuclear gene *ApoB* (ΔCt) and compared against the experimental conditions considered as control (ΔΔCt) to subsequently obtain the relative expression (2^-ΔΔCt^) as the mtDNA expression level compared to the control condition (mtDNA/nDNA).

### Expression profiling of genes related to mitochondrial bioenergetics by RT-qPCR microarrays

cDNA was synthesized from 500 ng of RNA total with the iScript cDNA Synthesis kit (Bio-Rad) at 25 °C for 5 min followed by 30 min at 42 °C and finally 5 min at 85 °C in a CFX96 thermocycler (Bio-Rad). Subsequently, the cDNA was mixed with iQ SYBR Green Supermix, and 25 µL was placed in each well of the "Mitochondrial Energy Metabolism" microarray for rats (Qiagen). Then, the reactions of microarray were amplified by qPCR on a CFX96 thermocycler (Bio-Rad) with the following parameters: 1 cycle of 95 °C for 3 min followed by 40 cycles of 95 °C for 15 s, 60 °C for 1 min and 70 °C for 30 s. The data were obtained with the CFX manager software (Bio-Rad). Consecutively, the mRNA fold change expression levels in liver tissue versus the control condition were calculated by the comparative Ct method.

### Electron microscopy

The liver tissue samples were fixed with glutaraldehyde (6%) and stained with osmium tetroxide (1% in a phosphate salt buffer). Once treated, the samples were dehydrated by passing through solutions with different percentages of alcohol, starting with a 70% preparation and ending with 100%, leaving the tissue for 20 to 30 min in each wash, maintaining a temperature of 4 °C. Once the dehydration was finished, it was infiltrated in propylene oxide and later in an epoxy resin, where it was kept for 2 h. After the resin preparation and the time required for polymerization, the blocks generated were cut and observed under an electron microscope.

### Identification of mitochondrial supercomplexes by blue native polyacrylamide gel electrophoresis (BN-PAGE)

Mitochondrial fractions were obtained as described above. Then, 1 mg of mitochondria was solubilized with 90 µL of sample buffer (ɛ-aminocaproic acid 750 mM, bis–tris 50 mM, pH 7.0), protease inhibitors (Complete 1X and PMSF 1 mM) and digitonin at a concentration of 2 mg/mg of sample. After the mixture was prepared, the samples were shaken at 4° for 30 min. Once the time had elapsed, the samples were centrifuged at 23,680 rpm for 1 h and 15 min at 4 °C. The supernatant was recovered, and protein was quantified by the Bradford assay. Consecutively, 5% Coomassie Blue (final concentration in-gel of 0.25%) was added to the samples. Acrylamide gradient gels were prepared from 4 to 10% (49.5% Acrylamide, 1.5% bis-acrylamide); samples were placed on the gels, and cathode buffer 1 (50 mM Tricine, 15 mM Bis–Tris and 0.02% Serva Coomassie Blue), anode buffer (50 mM Bis–Tris, pH 7.0 adjusted with HCl) was added to the electrophoresis chamber. The gel was run at 15 mA per gel. The run was stopped when the front was in the first third of the gel, and the cathode buffer was changed to cathode buffer 2 (Tricine 50 mM, Bis–Tris 15 mM, and Serva Coomassie Blue 0.002%). Finally, the run continued for approximately 1 h and 20 min. Stoichiometry was determined by analyzing the activity of complex I, II, IV and V compared against the CI band activity; the results were compared with the stoichiometry reported in literature^52–54^.

### Clear native gel electrophoresis (CN-PAGE)

1 mg of isolated liver rat mitochondria were solubilized with 10% lauryl maltoside, shaken for 30 min at 4 °C, and then centrifugated at 17,500 rpm by 75 min 4 °C. The protein concentration was determined by the Bradford method, and 0.1–0.25 mg protein per well was loaded in 4–12% polyacrylamide gradient gels; therefore, the same amount of mitochondrial protein was added for each condition. For clear native electrophoresis was used 0.01% lauryl maltoside and 0.05% sodium deoxycholate were in the cathode buffer. Gels were run for one hour at 15 mV/gel in a Bio-rad electrophoresis chamber^55^. In-gel NADH: NBT oxidoreductase activity (Complex I) was determined by incubating the native gels in 10 mM Tris (pH 7.0), 0.5 mg/mL nitrotetrazolium blue chloride (NBT), and 1 mM NADH. In-gel succinate: NBT oxidoreductase activity (Complex II) was defined by incubating the native gels in 10 mM Tris (pH 7.0), 0.5 mg/mL NBT and 1 mM succinate. In-gel cytochrome c oxidase activity (Complex IV) was determined using 20 mg/mL diaminobenzidine (DAB) and 10 mg/mL cytochrome c in 50 mM phosphate buffer pH 7.4. In-gel ATPase activity (Complex V) was measured by incubating the CN-gel in 270 mM glycine and 35 mM Tris buffer pH 8.4 and added 0.2% Pb(NO_3_)_2_, 14 mM MgSO_4_ and 8 mM ATP^56^.

### Identification of mitochondrial phospholipids

Mitochondrial proteins (2 mg) were used to determine mitochondrial phospholipids. Folch's reagent (chloroform: methanol 2:1) was added at a ratio of 1 mL/mg of sample; the samples were left for 24 h. Once the time elapsed, the samples were transferred to a new container filtering the content. After the filtration and change of container, the sample was left in dialysis for 24 h. The sample was transferred to a 5 mL tube, and 4 mL of methanol was added and homogenized; the sample was left until the methanol evaporated. Finally, 100µL of methanol was added to the sample in the tubes and homogenized. With the sample resuspended and cooled, thin layer chromatography (TLC aluminum silica gel 60F F_254_) was performed, cutting the appropriate size according to the number of samples. Chloroform: methanol: 12 mM MgCl_2_ in a 65: 25: 4 proportion was used as the mobile phase, and 10% of cupric sulfate in 8% ortho-phosphoric acid was used as revealing^57^. Phosphatidylcholine (PC), sphingomyelin (SM), and lysophosphatidylcholine (LPC) were identified according to previous reports; meanwhile, cardiolipin 5.1 mg/mL in methanol was used as standard^57^.

### Mitochondrial membrane potential

The mitochondrial membrane potential was determined according to Baracca et al. (2003). A calibration curve was done by plotting rhodamine 123 fluorescence against the membrane potential calculated through the Nerst equation^58^.

### Complex I and II activity

The 2,6-dichloro-indophenol reduction was used to determine complex I and II activity in mitochondria isolated from the liver. Briefly, 1 mg/mL of mitochondrial protein was added to a medium containing 0.25 M sucrose, 1 mM EDTA, 10 mM Trizma base, pH 7.3, 0.5 mM KCN, 1 µM antimycin, 10 mM glutamate, and 10 mM malate as the substrate for complex I activity or 10 mM succinate as a substrate for complex II activity, and 152 µM rotenone (for the complex II activity determination). The absorbance was followed at 600 nm, and the activity was calculated using the 2,6-dichloro-indophenol extinction coefficient (21 mM^−1^ × cm^−1^).

### Complex III activity

Enzymatic assay of cytochrome c reductase was used to determine complex III activity in mitochondria isolated from liver samples. Initially, 1 mg/mL of mitochondrial protein was incubated in a reaction mix solution containing 30 mM glycylglycine, 0.033% cytochrome C (CAS No.:9001-16-5, Sigma-Aldrich), 0.24 mM B-NADH, 0.5 mM KCN for the cytochrome c oxidase inhibition and potassium buffer pH 8.5. Then, absorbance was followed at 550 nm every 5 s for 8 min. Ultimately, the activity was calculated using the millimolar extinction coefficient between oxidized and reduced cytochrome c, at pH 8.5 = 21.

### ATP levels determination in liver tissue

The levels of ATP were determined using the ATP Assay Kit (Colorimetric/Fluorometric) (Cat. ab83355, Abcam). The instructions provided by the manufacturers were followed.

### Data analysis

Data were plotted as the mean ± standard error of the mean (SEM) and analyzed by one-way analysis of variance (ANOVA) with Bonferroni's post hoc for multiple comparisons for those presenting statistical evidence of a normal distribution. For non-normally distributed results, a nonparametric analysis using Krustal-Wallis with Dunn's post hoc was applied; graphs were represented as median ± interquartile range (IR). Statistical differences were represented as (*) *P*-value < 0.05, (**) *P*-value < 0.01, and (***) *P*-value < 0.001 for each test applied.

### Supplementary Information

Supplementary Information.

## Data Availability

All relevant data are included in the manuscript or supplementary material. Any additional request can be directed to the corresponding authors.
