# Supplementary material for: An adenosine derivative promotes mitochondrial supercomplexes reorganization and restoration of mitochondria structure and bioenergetics in a diethylnitrosamine-induced hepatocellular carcinoma model
Source: Sci Rep. 2024 Mar 15;14:6348. doi: 10.1038/s41598-024-56306-9 (PMC10943223; doi:10.1038/s41598-024-56306-9)
Supplement: Supplementary file 1 — Supplementary Information. [file 41598_2024_56306_MOESM1_ESM.docx]

**Supplementary Information**

**Supplementary Figure 1**


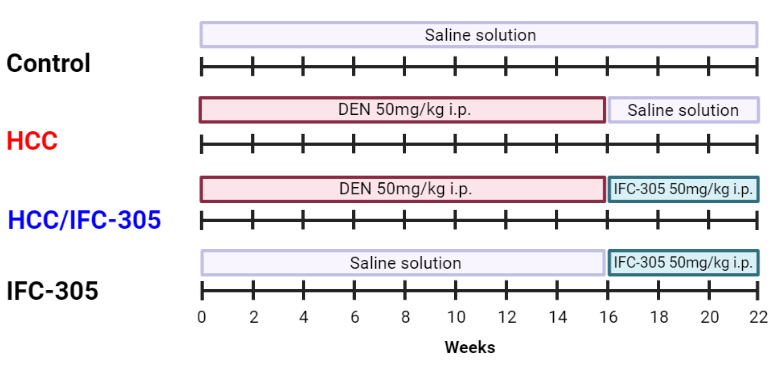


**Supplementary Figure 1. Sequential cirrhosis-hepatocellular carcinoma model in rats.** General protocol of administration of saline, DEN, or IFC-305 in the various treatment schedules; DEN is administered at a dose of 50 mg/kg i.p. 1 time per week; IFC-305 is administered at a dose of 50 mg/kg i.p. 5 times per week. Control and IFC-305 groups (n = 6); HCC and HCC/IFC-305 (n = 20).

**Supplementary Figure 2**


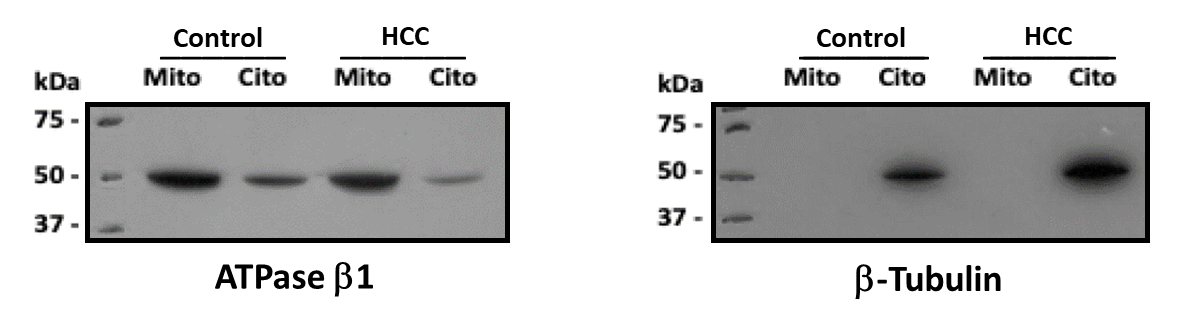


**(A)**

**(B)**

**ATPase β1**

**α-Tubulin**

**Supplementary Figure 2. Purification of cellular fractions.** Western blot of **A)** mitochondrial fraction of β1-ATP synthase and **B)** cytosolic fraction using α-tubulin as a specific protein.

**Supplementary Figure 3**


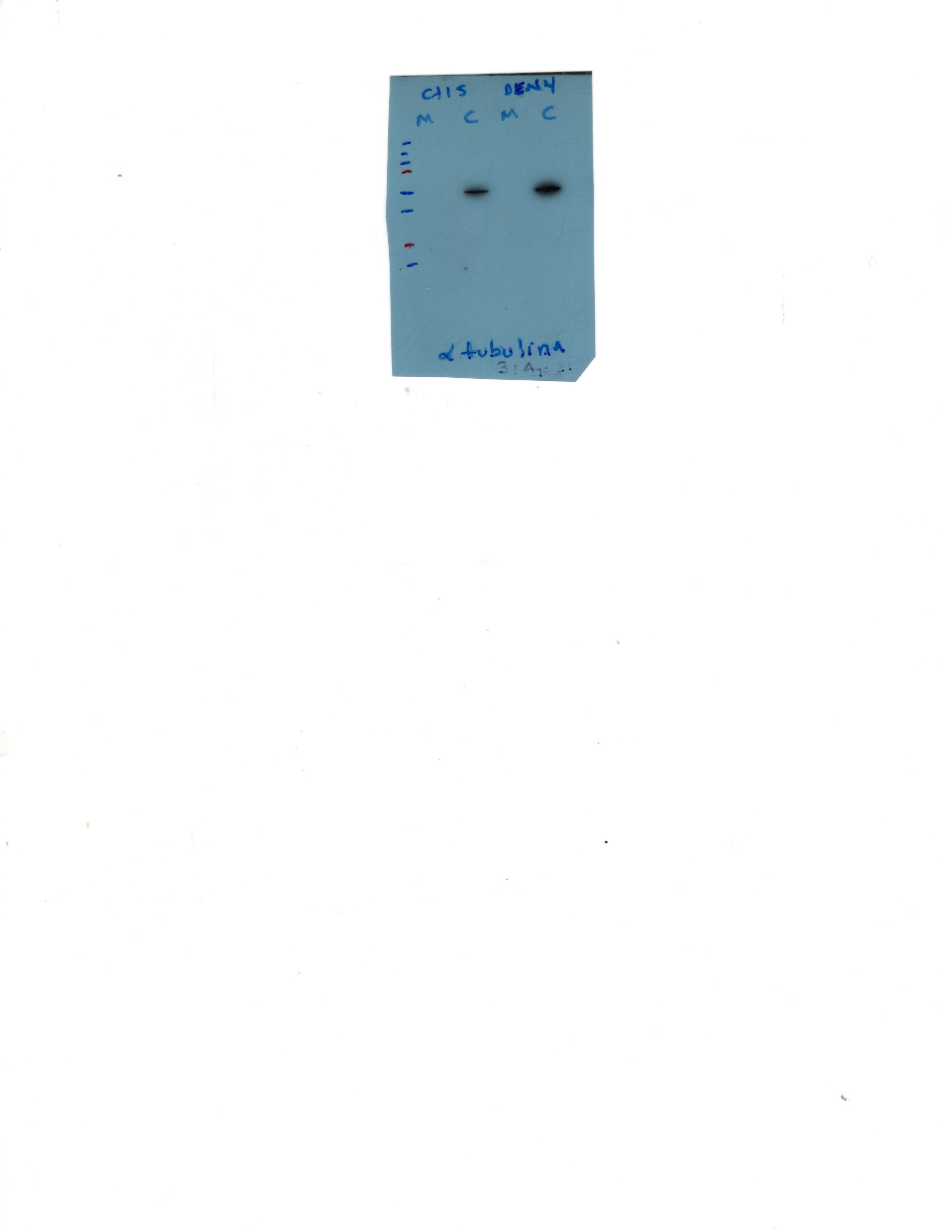


75

50

37

25

10

100

150

**B)**

**A)**

**1**

**2**

**1**

**2**

kDa

kDa


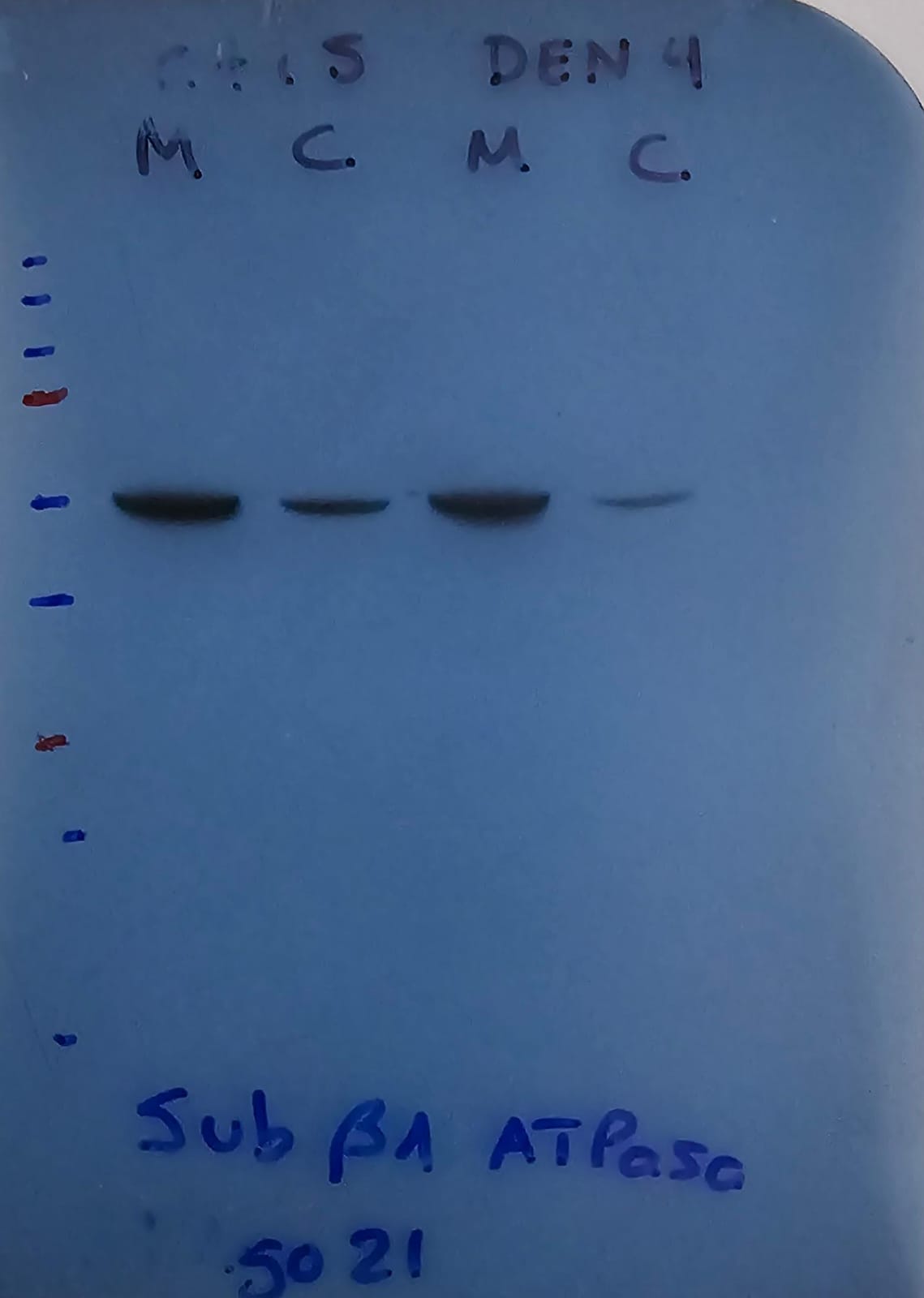


75

50

37

25

10

100

150

**Supplementary Figure 3. Images of original representative uncropped Western blots for Supplementary Figure 2.** Uncropped Western blot images are shown that correspond to supplementary figure 2 in **A)** mitochondrial fraction of β1-ATP synthase (Anti-alpha Tubulin antibody [DM1A], abcam, No. ab7291) and **B)** cytosolic fraction using α-tubulin Anti-ATPB antibody [3D5, abcam, No. ab14730) as a specific protein detection. Number 1 corresponds to the control group, and number 2 to the HCC group: letter M=mitochondrial fraction and C=cytosolic fraction.

**Supplementary Figure 4**

**(C)**

**(B)**

**Complex IV Activity**

**Complex II Activity**

**Complex I Activity**

**(A)**


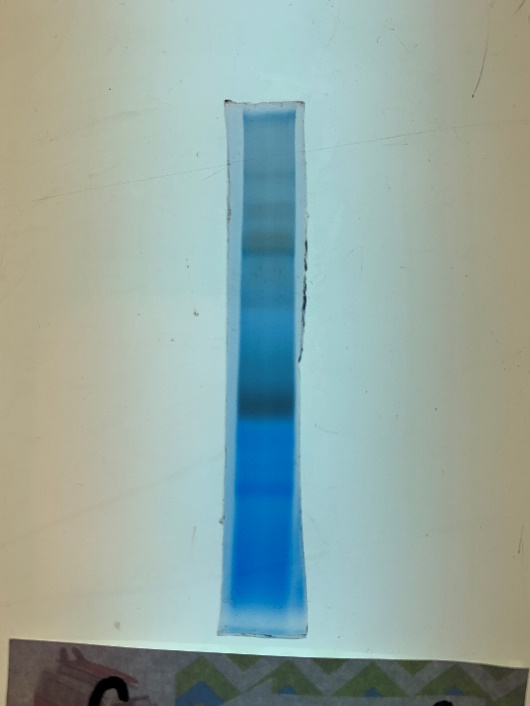

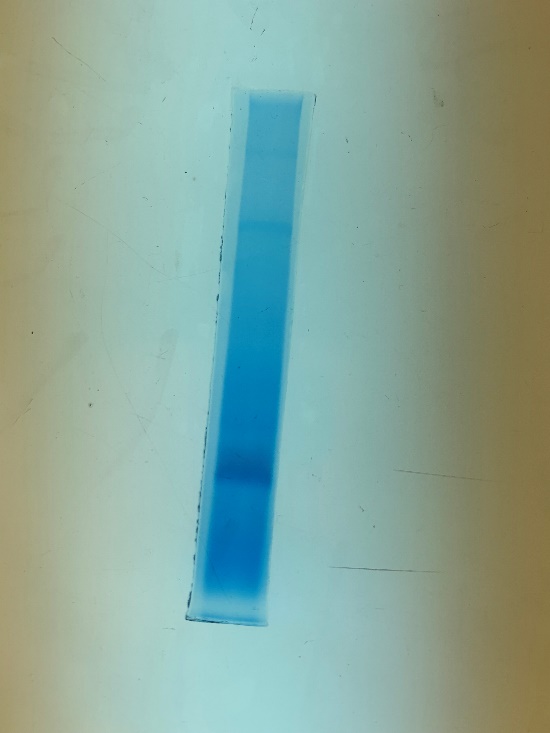

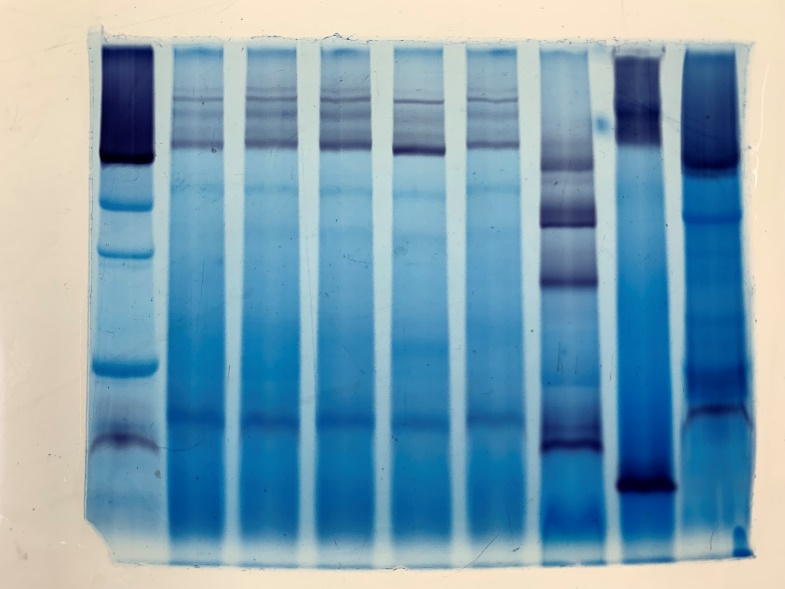


MW

(kDa)

500 -

750 -

1,000 -

200 -

130 -

**C5**

**C4**

**C3**

**C1**

**C2**

**BH**

**
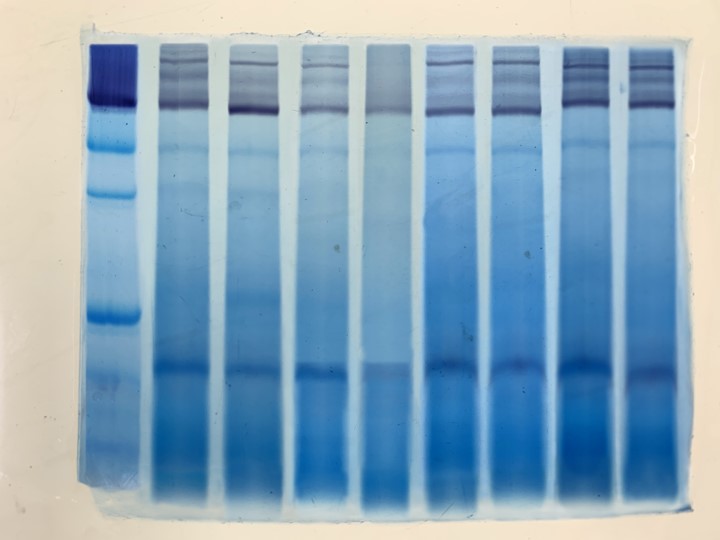

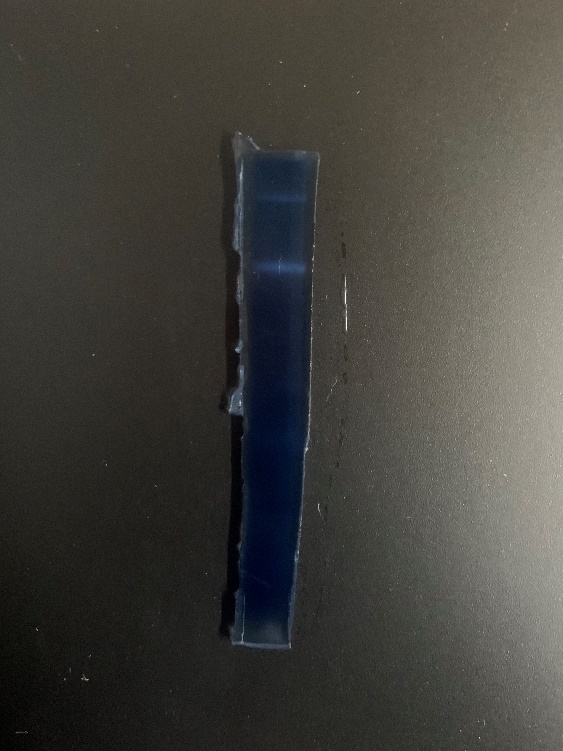
**

**(E)**

**(D)**

**hmwSC Complex I Activity**

**Complex V Activity**

**8**

**7**

**6**

**5**

**4**

**3**

**2**

**1**

**BH**

**Supplementary Figure 4. Original images of BN-PAGE for Complex I, II, IV, and V activity**. **A)** Complex I activity analysis: the first lane corresponds to bovine heart (BH) mitochondria as a positive control, and lines 2-6 represent liver mitochondria of the five rats of the experimental control group (C1 to C5). **B)** Representative line for complex II activity. **C)** Representative line for complex IV activity. **D)** Representative line for complex V activity. **E)** Representative image for Complex I activity in liver mitochondria samples obtained of DEN-induced HCC model experimental groups: bovine heart (BH), 1-2 (Control), 3-4 (HCC), 5-6 (HCC/IFC-305), 7-8 (IFC-305). The activity of complexes I, II, IV, and V (B-D) was determined separately because different substrates and buffer solutions are used to determine the activity of complexes, but the representative lines included correspond to the same BN-PAGE. The position and possible stoichiometry of the hmwSCs were determined by literature and comparing the activity of each complex against the complex I activity.

**Supplementary Figure 5**

**(A)**

**TLC for mitochondrial phospholipids**


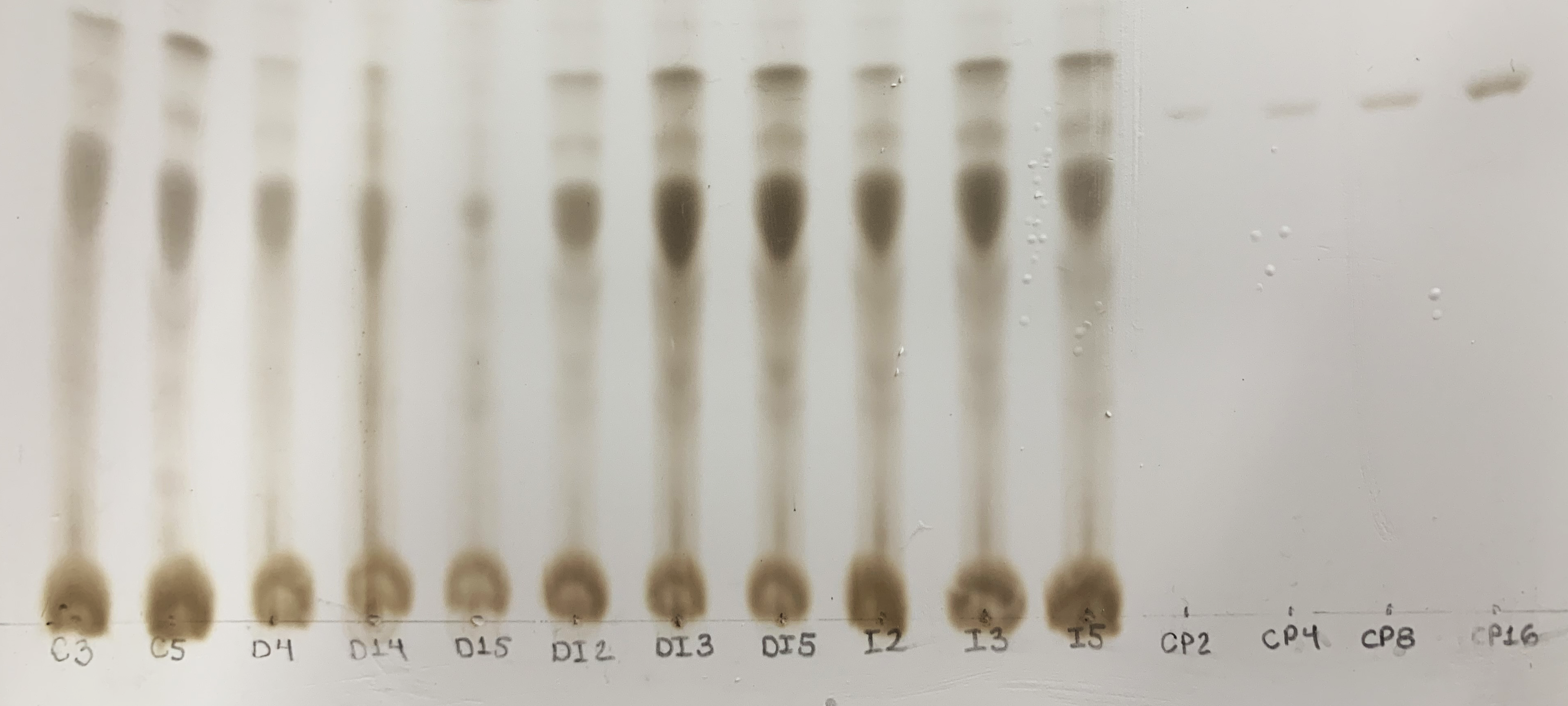


**C**

**D**

**DI**

**I**

**CP**

**Supplementary Figure 5. Original image of Mitochondrial phospholipids determination by TLC.** The representative image represents a TLC from mitochondrial phospholipids. Each lane corresponds to samples control (C), HCC (D), HCC/IFC-305 (DI) and IFC-305 (I). The last lanes correspond to increasing concentrations of cardiolipin (CP).

**Supplementary Figure 6**


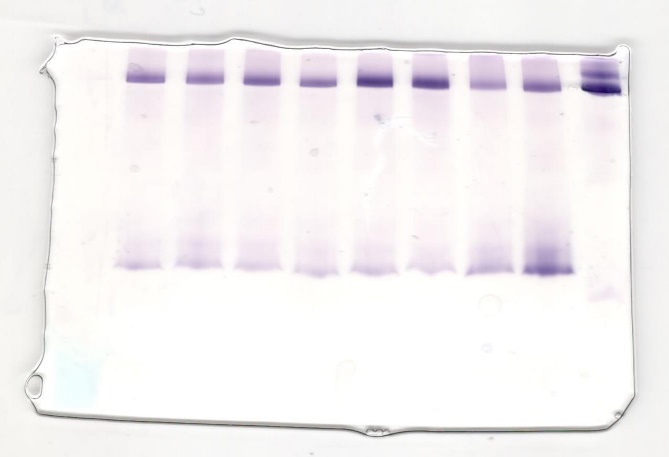

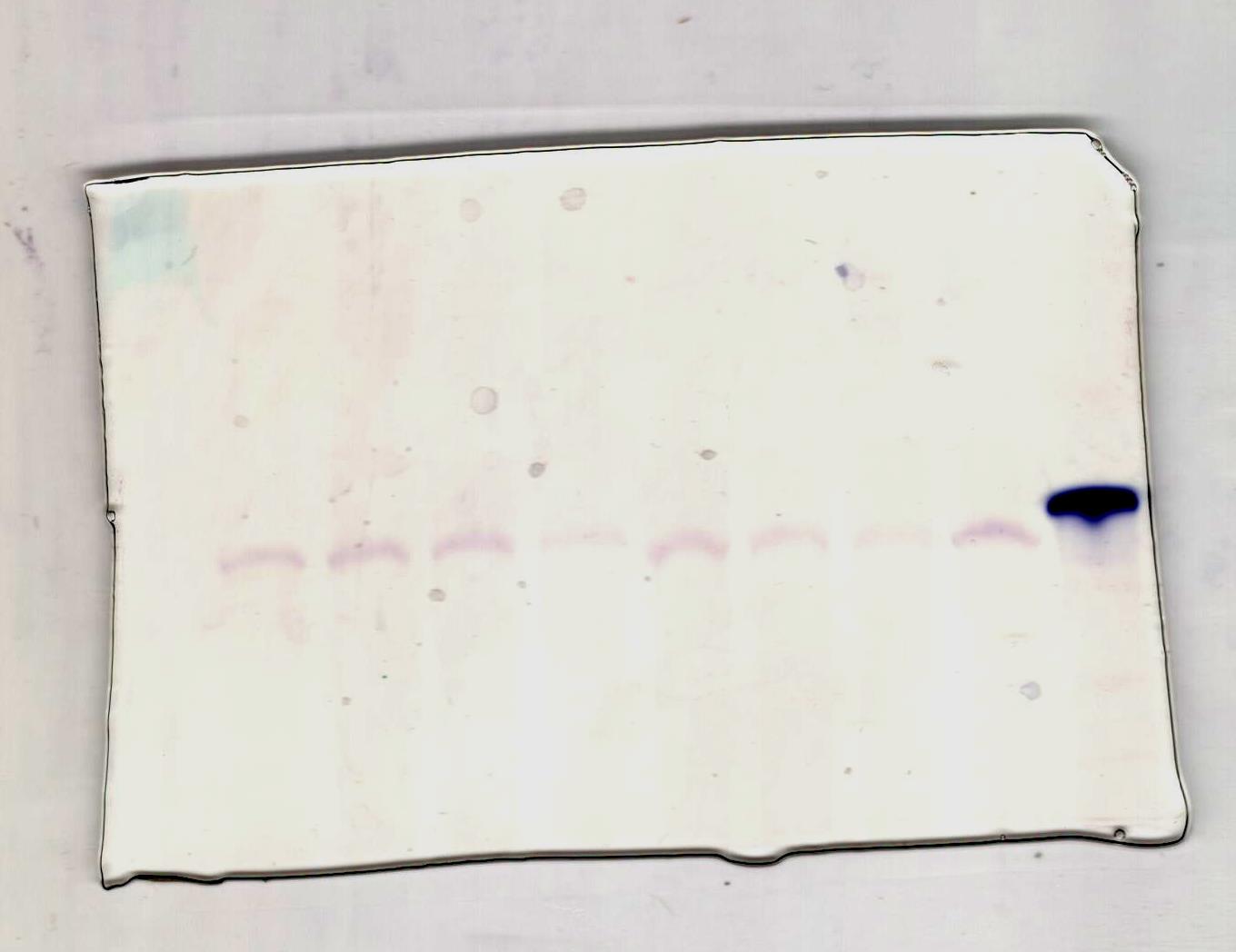

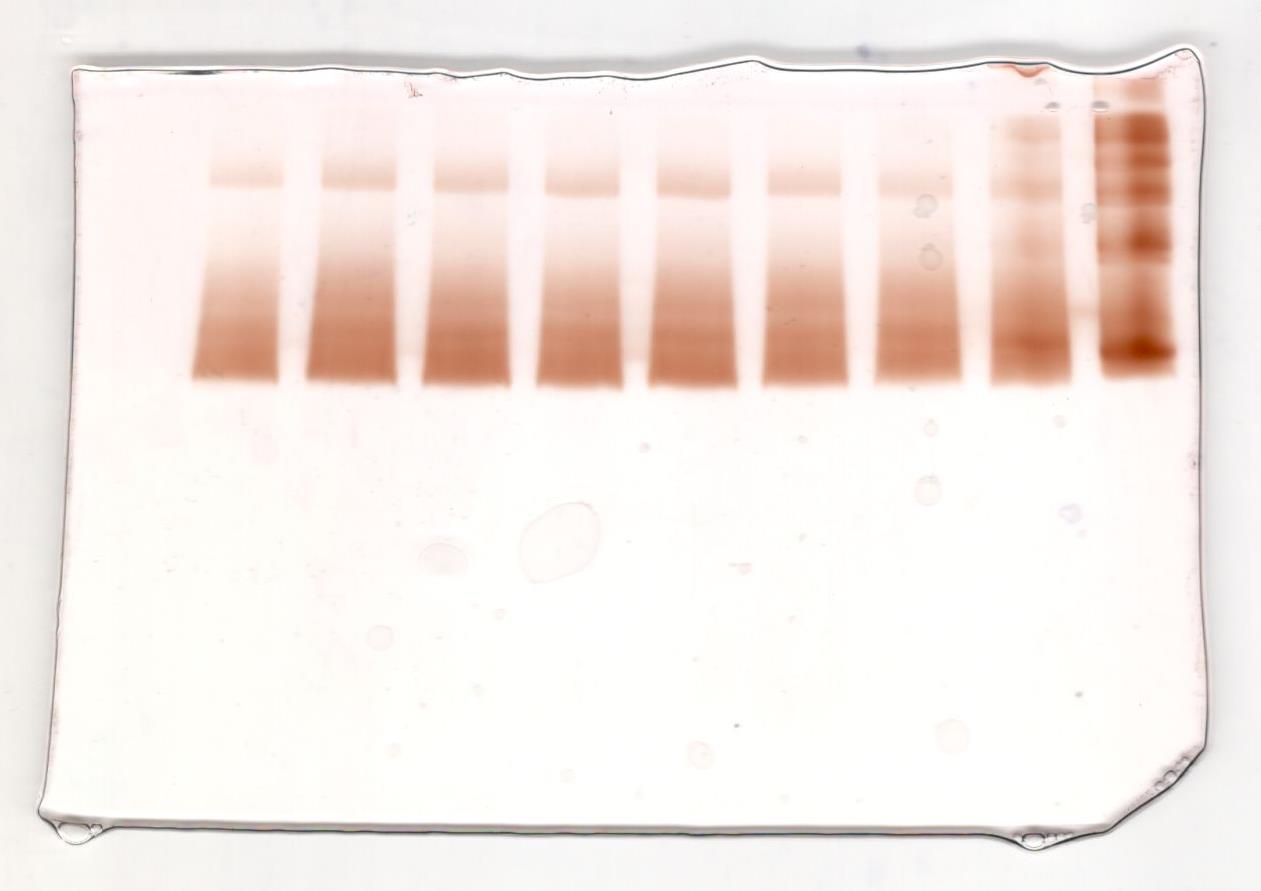

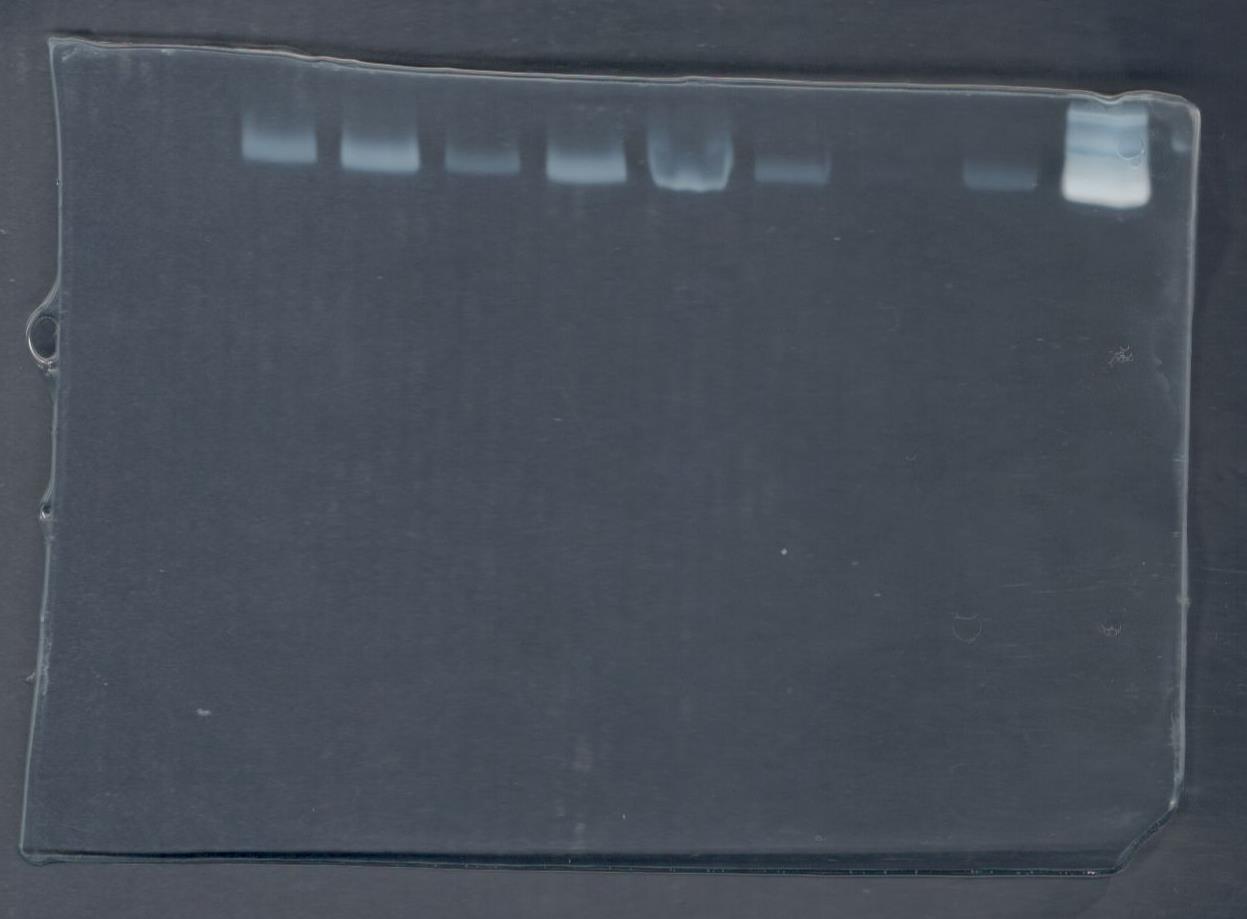


**A)**

**B)**

**C)**

**D)**

BH

BH

BH

BH

1

2

3

4

1

2

3

4

CI

1

2

3

4

1

2

3

4

1

2

3

4

1

2

3

4

1

2

3

4

1

2

3

4

CII

CIV

CV

**Supplementary Figure 6. Original images of representative uncropped CN-PAGE for Figure 6.** Uncropped clear native gels that correspond to figure 6. **A)** Mitochondrial complex I activity, **B)** Mitochondrial complex II activity, **C)** Mitochondrial complex IV activity, and **D)** Mitochondrial complex V activity. BH= bovine heart as positive control, 1=control, 2=HCC, 3=HCC/IFC-305, and 4=IFC-305.

**Supplementary Table 1**

|  | | |
| --- | --- | --- |
|  | Sequence | |
| Gen | Forward | Reverse |
| *Gapdh* | 5'- GTCTACTGGCGTCTTCACC -3' | 5'- CCACGATGCCAAAGTTGTCA -3' |
| *Afp* | 5'- AGCGCATCCATTTCCTTCCT -3' | 5'- TTCATTGCAGCCAACGCATC -3' |
| *Mki67* | 5'- AAGAGAGCATCCATCAGCCG -3' | 5'- CCCAAAATCTCTGAGGCGGT -3' |
| *Gpc3* | 5'- ATCCAGCCGAAGAAGGGAAC -3' | 5'- CAGCACAGGGTGTCGTTTTC -3' |
| *Col1a1* | 5'- ATGGTGAGACGTGGAAACCTG -3' | 5'- AGAAAGGACAGCACTCGCC -3' |
| *Cox7a* | 5'- GGTGACTGAGAAGGCTGACG -3' | 5'- CCATGTAGAGGGCGATCAGG -3' |
| *Uqcc3* | 5'-TGTTTGCCTTAGTGACCCCA -3' | 5'- AGGTGCCTTACAGATTCCCA -3' |
| *Higd2a* | 5'- TCTACACCAATCCAGAGGGC -3' | 5'- AGTAAAGGCCATAGGTGAGTG -3' |
| *Spg7* | 5'- ATGAAAGACCCTCTGAGCCC -3' | 5'- AAAACAAGACCCGTGACTGC -3' |
| *Yme1l* | 5'- GCGTTGCTGACCTATGAGAC -3' | 5'-TGGAAGTGTTGCAAAGGAGAC -3' |
| *Opa1* | 5'- TGTAGCCAGTCCAAGCAGAA -3' | 5'- ATTTCTCGTGGTCACCTGGT -3' |
| *Atp11a* | 5'- CAGCCTATTGAGGACGCTCG -3' | 5'- GTGTCCCACGTAGATGGTCC -3' |
| *Ndufa6* | 5'- CGGTGAAACAAGGACGGGAT -3' | 5'-TGACCAGGAGGTCAACCACT -3' |
| *Cox5b* | 5'- GTGGGCTGCATCTGTGAAGA -3' | 5'- GAAATGTTAAACCAGGGGTGGG -3' |
| *Cox1* | 5'- GCTGACTCGCTACACTACATG -3' | 5'- AGTGGGAATCAGTGGACGAA -3' |
| *Apob* | 5'- TCAAGTCCCTCCCTGAGTGC -3' | 5'- CATGGCCGATGGATGGGTAG -3' |
|  | | |

**Supplementary Table 1.- Oligonucleotides used for mRNA detection.** List of primers utilized in qPCR protocol to determine every gene expression level.
